# Using health economic modelling to inform the design and development of an intervention: estimating the justifiable cost of weight loss maintenance in the UK

**DOI:** 10.1186/s12889-022-12737-5

**Published:** 2022-02-12

**Authors:** Sarah E. Bates, Chloe Thomas, Nazrul Islam, Amy L. Ahern, Penny Breeze, Simon Griffin, Alan Brennan

**Affiliations:** 1 School of Health and Related Research, University of Sheffield, Sheffield, South Yorkshire UK; 2 Clinical Trial Service Unit and Epidemiological Studies Unit (CTSU), Nuffield Department of Population Health, University of Oxford, Oxford, UK; 3 MRC Epidemiology Unit, University of Cambridge, Cambridge, UK

**Keywords:** Health economic modelling, Weight loss maintenance, Behavioural intervention

## Abstract

**Background:**

There is a need to develop cost-effective weight loss maintenance interventions to prolong the positive impact of weight loss on health outcomes. Conducting pre-trial health economic modelling is recommended to inform the design and development of behavioural interventions. We aimed to use health economic modelling to estimate the maximum cost per-person (justifiable cost) of a cost-effective behavioural weight loss maintenance intervention, given an estimated intervention effect for individuals with: i) a Body Mass Index (BMI) of 28 kg/m^2^ or above without diabetes and ii) a diagnosis of type 2 diabetes prescribed a single non-insulin diabetes medication.

**Methods:**

The School for Public Health Research Diabetes prevention model was used to estimate the lifetime Quality-adjusted life year (QALY) gains, healthcare costs, and maximum justifiable cost associated with a weight loss maintenance intervention. Based on a meta-analysis, the estimated effect of a weight loss maintenance intervention following a 9 kg weight loss, was a regain of 1.33 kg and 4.38 kg in years one and two respectively compared to greater regain of 2.84 kg and 5.6 kg in the control group. Sensitivity analysis was conducted around the rate of regain, duration of effect and initial weight loss.

**Results:**

The justifiable cost for a weight loss maintenance intervention at an ICER of £20,000 per QALY was £104.64 for an individual with a BMI of 28 or over and £88.14 for an individual with type 2 diabetes. Within sensitivity analysis, this varied from £36.42 to £203.77 for the former, and between £29.98 and £173.05 for the latter.

**Conclusions:**

Researchers developing a weight loss maintenance intervention should consider these maximum justifiable cost estimates and the potential impact of the duration of effect and initial weight loss when designing intervention content and deciding target populations. Future research should consider using the methods demonstrated in this study to use health economic modelling to inform the design and budgetary decisions in the development of a behavioural interventions.

**Supplementary Information:**

The online version contains supplementary material available at 10.1186/s12889-022-12737-5.

## Introduction

Overweight and obesity is a risk factor for several negative health outcomes including cardiovascular disease (CVD), diabetes and cancer [1]. Behavioural weight management programmes have been associated with significant weight loss [2] and can even result in remission from type 2 diabetes [3] but there is evidence that, on average, individuals regain weight loss by 5 years post-treatment [4]. Furthermore based on a large observational study, only 21% of individuals are successful at maintaining weight loss, defined as losing at least 10% of their body weight and maintaining this weight loss for at least one year [5]. While moderate reductions in weight have positive benefits for individuals who are overweight or obese and for those who have type 2 diabetes even if weight loss is regained [6–8], weight loss maintenance is required to maintain full improvements in risk reduction. For example, individuals who lost 8–20% of their initial body weight and maintained this for 4 years (regained less than 3% of initial body weight) in a randomised control trial of a behavioural intervention achieved sustained improvements in blood glucose (HbA_1c_), systolic blood pressure (SBP) and cholesterol, all biomarkers linked with health outcomes [9]. Thus, there is a need to develop cost-effective weight loss maintenance interventions in order to prolong the positive impact of weight loss on health outcomes [10].

Conducting pre-trial health economic modelling is recommended to estimate the likelihood of cost-effectiveness, inform decision about whether a trial is justified, and identify potential improvements to the intervention (9). Using an estimated intervention effect based on previous research, a maximum cost-per-person (justifiable cost) can be estimated at which the intervention would remain cost-effective given a certain incremental cost-effectiveness ratio (ICER). This can be compared to expected costs to ensure that an intervention is not predicted to incur a cost at which it is unlikely to be cost-effective. Pre-trial modelling has been conducted previously; for example Asaria et al. (2016) used a health economic model to estimate the annual costs at which interventions with varying impacts on cardiovascular risk would be cost-effective for individuals with different risk profiles [11] and pre-trial modelling was used to inform the design of a fall-prevention intervention and trial [12]. However, these studies were either based on hypothetical, rather than intervention-specific, risk changes (10) or based on the results from a pilot trial (11) and so is not a method that can be use before a pilot trial has taken place. The aim of this analysis was to use a health economic model to determine the justifiable cost of a behavioural weight loss maintenance intervention compared to no intervention in two populations; i) individuals with a Body Mass Index (BMI) of 28 kg/m^2^ or above without diabetes and ii) individuals with a diagnosis of type 2 diabetes prescribed a single non-insulin diabetes medication.

## Methods

The reporting of this study followed the 2013 Consolidated Health Economic Evaluation Reporting Standards guidelines [13].

### SPHR diabetes prevention model

The School for Public Health Research (SPHR) Diabetes prevention model has been used to assess the cost-effectiveness of diabetes prevention interventions [14–16]. For this study we use version 3.3 of the model and full detail of the model background, methods, assumptions and parameters is in the Additional files 2 and 3.

The SPHR models is an individual patient level model in which the baseline characteristics of an individual are used to estimate annual changes in metabolic risk factors and the risk of related diseases. This model was used because it enables change in BMI to be modelled, trajectories of BMI and other metabolic factors to vary among individuals and estimates of the impact of weight loss and weight loss maintenance on a range of health conditions including CVD, type 2 diabetes, osteoarthritis, and depression. The model structure is shown in Additional file 2, Fig. 1. Each year changes in metabolic factors, namely BMI, HbA1c, SBP and total cholesterol, occur depending on the individual baseline characteristics including age, sex, ethnicity, smoking, family history of CVD, and family history of type 2 diabetes. Associations between the trajectories of the metabolic risk factors were based on latent growth curve modelling analysis conducted on the Whitehall II prospective cohort study [17]. Change in glycaemia, SBP and total cholesterol are all conditional on change in BMI.

**Fig. 1:**
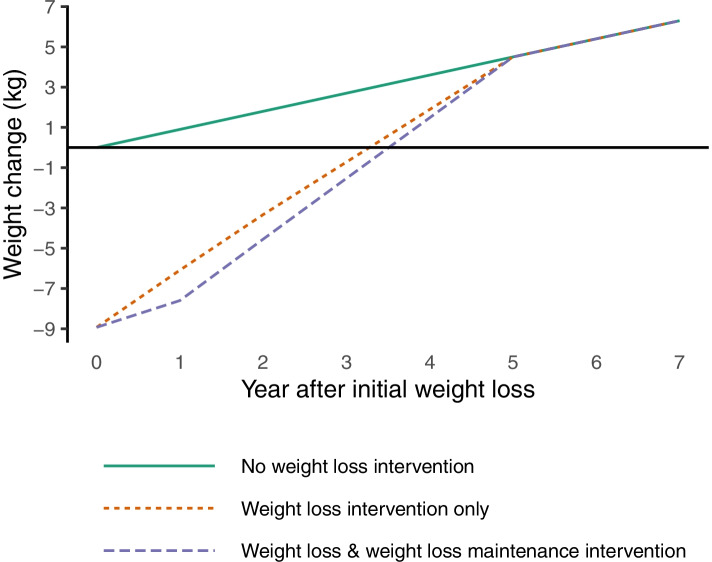
Simulated trajectories of weight change post initial weight loss

These metabolic factors then contribute to the risk of an individual patient experiencing a disease or related complications. At GP visits, an individual in the model may be diagnosed with diabetes, hypertension, and dyslipidaemia. GP attendance is conditional on age, sex, BMI, ethnicity and health outcomes (heart disease, depression, osteoarthritis, diabetes, stroke, cancer) based on the South Yorkshire Cohort study [18]. Individuals can also experience cancer (breast or colon), osteoarthritis and depression, CVD events (angina, myocardial infarction (MI), stroke, or transient ischemic attack (TIA) and diabetes related complications (renal failure, amputation, foot ulcer, and blindness) based on risk equations described in section 7 of Additional file 2. Many of the diagnoses and events in the model are conditional on BMI. It contributes to the risk of the first cardiovascular events as part of the QRISK2 prediction model [19]. This is a validated algorithm to identify individuals at high risk of cardiovascular disease. Subsequent cardiovascular events are conditional on the nature of the first event. Incidence of breast and colorectal cancer were estimated from the European prospective investigation of cancer (EPIC) cohort [20] and based on a large meta-analysis including 221 prospective observational studies [21], a risk adjustment was included such that individuals with a high BMI have a higher probability of the cancer diagnosis. Osteoarthritis was also conditional on BMI; this was based on a stakeholder discussion and a longitudinal analysis based in Italy as there were no appropriate UK studies available [22]. A diagnosis of diabetes was dependent on blood glucose (HbA_1c_), the trajectory of which is associated with BMI and, of the diabetes-related complications, neuropathy (ulcer and amputation) was conditional on BMI based on the UKPDS outcomes model v2 [23]. Depression was not conditional on BMI however it was assumed that a diagnosis of diabetes and/or cardiovascular disease increased the incidence of depression for individuals who did not have depression at baseline based on two US cohort studies [24, 25]. Depression was not a causal factor for any health outcomes in the model.

The consequences of interventions are measured in Quality Adjusted Life Years (QALYs), as recommend by the National Institute for Health and Care Excellence (NICE) [26], based on the EQ-5D-3L, and costs/savings in pounds sterling. The model has an annual cycle length and a lifetime horizon as weight loss and maintenance have the potential to impact long-term health outcomes. The setting is primary care in England, UK and a healthcare perspective (National Health Service (NHS) in England) was used. This includes cost healthcare costs incurred by the NHS and excludes any costs incurred by the patient such as travel and time costs associated with the intervention. Both costs and QALYs were discounted at an annual rate of 3.5% as recommended by NICE [26].

### Populations

The analyses were conducted for two separate populations; i) individuals with a BMI of 28 kg/m^2^ or above without diabetes and ii) individuals with a diagnosis of type 2 diabetes prescribed one non-insulin diabetes medication. These populations were chosen as they are at high risk of negative health impacts, have the potential to respond to early intervention (i.e. before developing diabetes, or diabetes dependent on insulin or several medications) and were likely target populations for this type of intervention [27]. The baseline characteristics of both populations can be found in Supplementary Table 1.

For population (i), the baseline data on individuals was obtained from Health survey for England (HSE) 2014 [28], which is representative of the population of England and includes clinical risk factors including HbA1c, SBP, BMI and cholesterol and health outcomes. The population of interest was defined as adults with a BMI of 28 kg/m^2^ and over (prior to initial weight-loss), based on previous studies in which this was a criteria for referral to a weight management programme by a GP [29], and with a HbA_1c_ below 6.5% (the criteria used for a diabetes diagnosis). Children aged under 18 and adults with a diagnosis of diabetes were excluded. Within the final sample (*n* = 2738), a subgroup of individuals with an HbA_1c_ of 6–6.49% were examined separately (*n* = 322) as this criteria is used to identify individuals at higher risk of diabetes [30].

For population (ii), HSE only included a small number (approximately 400) of individuals with diabetes and thus would be unlikely to represent the diabetic population well and has little information about the diabetes diagnosis such as time of diagnosis and treatment. For this population, the THIN (The Health Improvement Network) 2014 dataset [31] was used which has a large number of individuals with diabetes. Of the 3.7 million individuals from 427 GP practices, 131,000 had type 2 diabetes. The time since diagnosis and treatment prescribed was also available for this dataset alongside BMI, HbA1c, cholesterol, and SBP and demographic factors such as age, gender, and ethnicity. A baseline population was created by sampling from the summary statistics of this data by using a multivariate distribution using the mean estimates and covariances between these variables. Individual patient level data was not available due to restrictions on the use of this at the time of analysis. Although individual data is preferable, this method enabled the use of a baseline population that was representative of individuals with diabetes. The sample was not restricted by time spent on this medication but those on more than one anti-diabetic mediation or on insulin were excluded. A subgroup analysis for those with a BMI of 28 or above was also included based on previous studies in which this was a criteria for GP referral to a weight management programme [29].

The structure and assumptions in the model remained the same for both baseline populations. The model enabled different health trajectories for those with and without diabetes which enables the model to be flexible to both populations. For example, for individuals without diabetes, the trajectory of HbA1c was estimated based on an analysis of the Whitehall II dataset [17] however for those with diabetes, the trajectory is estimated using the UKPDS outcomes model [32], a population of individuals newly diagnosed with diabetes. Similarly, individuals with a diagnoses of diabetes are eligible for antihypertensive treatment at the threshold of a SBP of 140 mmHg compared to a threshold of 160 mmHg for participants without diabetes based on National Institute for Health and Care Excellence (NICE) guidelines (11).

### Intervention effect

The estimated effect of the intervention on weight has been obtained by examination of the literature. We conducted a random-effects meta-analysis of behavioural weight loss maintenance studies to estimate the expected effect of a weight loss maintenance intervention compared to no intervention (current standard care in the UK) after weight loss resulting from a behavioural intervention. Following the PRISMA process, relevant studies were screened from two previous systematic review and meta-analysis studies of weight loss maintenance interventions [4, 33] to identify those studies that met our pre-specified inclusion criteria. The inclusion criteria were chosen to reflect likely commissioning of services in the UK NHS and were informed by current practice and discussions with our stakeholder group comprising health economists, clinicians and researchers and lay members. Studies had to include adult participants with a BMI ≥ 25 kg/m^2^, who had lost ≥5% of their weight before starting the weight loss maintenance programme. Studies that required ≥10% initial weight loss to join the study or which solely recruited participants with a specific health condition were excluded as this population was deemed highly selective and not representative of the intended population. The intervention had to be a behavioural intervention including advice on diet and physical activity for the primary purpose of weight management. Interventions that used meal replacements and financial incentives were excluded as these interventions are unlikely to be widely commissioned in the UK NHS. Studies had to report weight outcomes ≥12 months from the start of the weight maintenance intervention. Only randomised controlled trials were included. We applied these inclusion and exclusion criteria to the two systematic reviews, which reported data from a total of 32 behavioural intervention arms from 20 studies [34–53]. Nine studies were excluded from our analyses for the following reasons: (a) inclusion criteria did not reflect the target population, [35, 42, 49, 53] (b) intervention included meal replacement or financial incentives [38, 46, 52] (c) primary purpose of the intervention was not weight management [51] or (d) did not report weight outcomes ≥12 months from the start of the weight maintenance intervention [37, 41].

Three analyses of the studies were undertaken. Firstly, fourteen intervention arms from nine studies [34, 39, 40, 43–45, 47, 48, 50] were included in a meta-analysis to estimate initial weight loss of participants that were eligible for a weight loss maintenance intervention. Second, fifteen intervention arms from ten studies [34, 36, 39, 40, 43–45, 47, 48, 50] contributed to the meta-analysis to estimate weight loss maintenance intervention effects at 12-month post-weight loss. Supplementary Fig. 1 in shows the nature of the control arm, and type of weight loss maintenance intervention for the studies included in this meta-analysis. All interventions targeted weight management through dietary and exercise advice but were varied in the method and duration of delivery, and control groups varied from no contact to in-person support. Third, two intervention arms from one study contributed to the estimates at 2-year post-weight loss [50] as this was the only eligible study that included a 2 year follow-up. The two interventions were unlimited access to an interactive technology–based intervention, and an intervention in which participants had monthly individual contact with an interventionist. Participants in the control group received printed diet and physical activity recommendations.

Table 1 shows the results of the random-effects meta-analysis; the initial weight loss before the weight maintenance intervention is estimated at 8.93 kg from an average initial weight of 89.76 kg, and individuals partaking in a weight loss maintenance intervention had an average regain of 1.33 kg by year 1 and 4.38 kg by year 2 compared to a regain of 2.84 kg by year 1 and 5.6 kg by year 2 in a control group. Forest plots comparing the active intervention with control group at 12- and 24-month follow-up are shown Supplementary Figs. 2 and 3. There was no evidence of an influence of individual studies on the overall estimates at 12 months (Supplementary Fig. 4). Influence plots were not generated for 24 months follow-up as only one study provided data at this time point. The revised Cochrane risk of bias tool for randomised trials [54] was used to assess the studies; four were low risk of bias [39, 40, 47, 50], 3 were high risk [34, 45, 48] and there were some concerns regarding the remaining three studies [36, 43, 44]. A sensitivity analysis in which the meta-analysis excluded the studies with a high risk of bias did not significantly impact the outcomes (Supplementary Table 2) There was moderate heterogeneity across studies in weight maintenance at 12 months (I^2^ = 59%, *P* = 0.002).

**Table 1:** Weight regain per annum: estimates from random-effects meta-analysis

	Weight maintenance intervention (*n* = 661)			Control (no intervention) (*n* = 383)			Difference between groups		
Year	N	Mean	95% CI^a^	N	Mean	95% CI^a^	N	Mean	95% CI
0	14	−8.93	(−9.49, − 8.36)	14	− 8.93	(− 9.49, − 8.36)			
1	15	1.33	(0.67, 1.99)	15	2.84	(2.01, 3.67)	15	−1.38	(−2.2, −0.55)
2	2	4.38	(3.64, 5.11)	2	5.6	(5.19, 6.02)	2	−1.23	(−1.96, −0.49)

N indicates total number of intervention arms; CI: Confidence intervals; estimates are in kg; ^a^95% CI of mean weight change. The weight for year 0 is the weight loss before weight maintenance intervention begins and the weight in year 1 and 2 is the weight regain per annum during weight maintenance intervention

#### Effect on weight trajectory beyond follow-up

In the absence of data on the longer-term weight trajectories, we made the conservative assumption that participants would return to baseline weight trajectory at some point. To determine when this point would be, the regain between years 1 and 2 was extrapolated linearly (assuming the same regain as between years 1 and 2 for each subsequent year), until the trajectory reached that of the simulated individual’s weight if they had never had the initial weight-loss intervention.). Both the control and treatment group returned to this original trajectory by 5 years (to the nearest full year) after the initial weight loss (Fig. 1). This aligns with research that indicates that on average participants regain weight loss after approximately 5 years [4]. Simulated individuals do not return to their baseline weight but the weight that they would have reached after 5 years in the SPHR model without the intervention. The initial weight-loss was simulated in year 0 at the start of the model, and then regained in subsequent years.

The trajectory of BMI is estimated in the health economic model but the weight change from the meta-analysis is in kg because it was the outcome measured in all studies. Therefore, the weight change in kg was converted to BMI change using the height of the simulated individual. In the absence of any data about the direct effects of the weight loss and weight regain on other metabolic factors, an indirect effect of the BMI change on HbA_1c_, SBP and cholesterol was modelled. Specifically, covariates from the analysis conducted on the Whitehall dataset were used to predict the change in the metabolic factors from changes in BMI in the population simulated [17] (detail in Additional file 2, page 18).

### Intervention costs

This analysis was conducted with the assumption that the proposed intervention would be funded for patient through primary care (i.e., the payer would be the NHS). This is already the case for some commercial weight loss and diabetes prevention programmes in the UK [55]. There is no fee charged to the individual receiving the interventions and patient borne costs (e.g., travel etc. are not included). Justifiable costs will be calculated for each person who has the intervention based on the assumption that all eligible individuals will participate in the intervention. It is assumed that all intervention costs will be incurred at time zero and so no discount rate is applied.

### Health economic modelling

For each run of the model, 20,000 eligible individuals were randomly sampled from the two baseline populations with replacement. As the aim of this analysis was to estimate a justifiable cost for a proposed intervention, the cost of the weight loss maintenance intervention was set to £0 within the model and the amount that could be spent on this intervention while remaining cost-effective was calculated using increasing maximum ICERs. For NICE, this is estimated to be between £20,000 and £30,000 per QALY [56] and therefore the cost per person at these ICER values were the targets for the analysis. Public health interventions often have a lower threshold because the benefits are further in the future, therefore the maximum cost of the intervention while being cost saving was also calculated. At this cost or lower, the cost savings as a result of the intervention is greater than the cost of the intervention.

### Sensitivity analysis

Sensitivity analysis was conducted on the duration of effect, the initial weight-loss and the rate of regain (Table 2). By duration of effect, we are referring to the amount of time between year 0 and the point at which the weight trajectories reach the trajectory they would have followed without any weight loss. Because the duration was estimated by extrapolating the regain from the first two years, in sensitivity analysis the impact of different durations (4–6 years) were examined (scenarios 1–3). A linear regain was assumed between the 2-year estimate of weight and the duration of effect (i.e., 4, 5 or 6 years). The rate of regain, the amount regained at year 1 and year 2, was varied using the 95% confidence intervals (CIs; scenarios 4 and 5). The weight loss that both groups achieved before entering either a weight loss maintenance intervention or control condition (no intervention) was also examined. The figure of 8.93 kg obtained from the meta-analysis is based on a target population of people who have lost ≥5% weight, which reflects the likely implementation of a weight loss maintenance programme in practice. We also examined a scenario in which there was not a minimum weight loss required to take part in the weight loss maintenance programme and examined the impact of a lower initial weight loss of 2.84 kg (scenario 6), based on average weight loss from a previous meta-analysis [2] of weight loss interventions that were applicable to UK primary care. An initial weight loss of 6.12 kg (scenario 7), which was the midpoint between the lower value of 2.84 kg and the base case value of 8.93 kg, was also tested. The regain was adjusted proportionally. These are represented graphically in Supplementary Fig. 5. Probabilistic sensitivity analyses were conducted to assess uncertainty within the model inputs using probabilistic sensitivity analysis with 5000 Monte Carlo simulations. The model parameters and uncertainty distributions are shown in Additional file 3.

**Table 2:** Scenarios modelled in sensitivity analysis

Scenario	Initial weight loss (*kg*)	Regain (*year 1, year 2)*	Duration of effect (*years*)	
			Control	Intervention
**Base case**	**8.96**	**1.33, 4.38**	**5**	**5**
1 [Duration]	8.96	1.33, 4.38	4	6
2 [Duration]	8.96	1.33, 4.38	5	6
3 [Duration]	8.96	1.33, 4.38	4	4
4 [Regain rate]	8.96	0.67, 3.64	5	5
5 [Regain rate]	8.96	1.99, 5.11	5	5
6 [Initial weight loss]	2.84	0.42,1.39	5	5
7 [Initial weight loss]	6.12	0.91, 2.99	5	5

## Results

### High BMI (≥ 28 kg/m^2^)

**Fig. 2:**
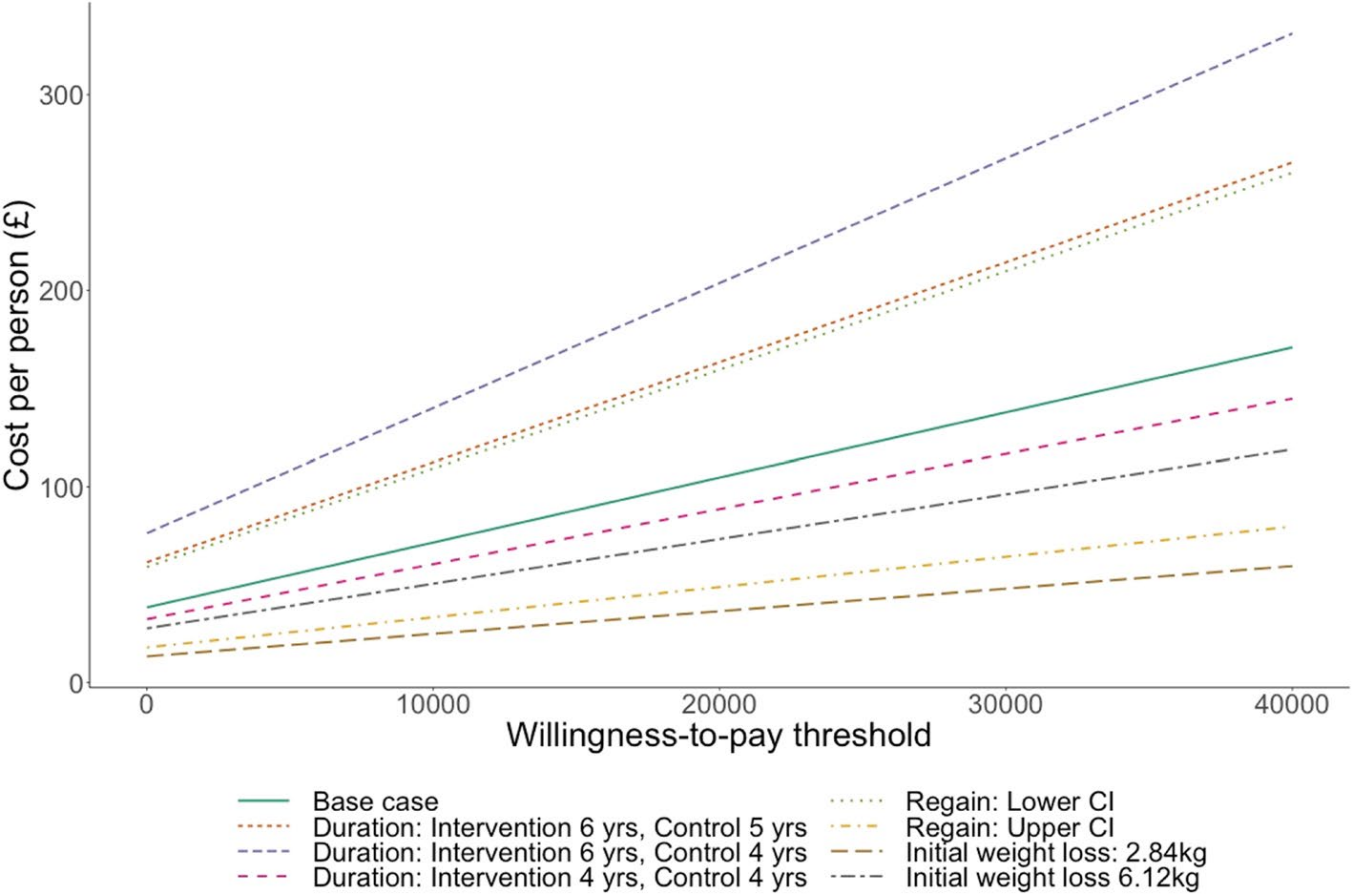
Justifiable cost per person: Base case and sensitivity analyses (BMI ≥28)

The estimated maximum amount that can be spent on an intervention while remaining cost-effective at increasing ICER values, with the assumption of the effect is detailed in Table 1, is shown in Table 3 and shown in Fig. 2. For ICERs of £20,000 and £30,000 per QALY, the maximum justifiable cost-per-person was £104.64 and £137.78 respectively assuming duration of effect of 5 years and health benefits accrued over the lifetime. For the subgroup that had a BMI ≥28 and an HbA_1c_ between 6 and 6.5%, the maximum justifiable cost-per-person was £158.88 and £209.81 respectively.

**Table 3:** Cost per person at incremental cost-effectiveness ratios of £20,000 and £30,000

Scenario		High BMI (≥ 28 kg/m^2^)		Type 2 Diabetes^a^	
		£20,000	£30,000	£20,000	£30,000
Base case		£104.64	£137.78	£88.14	£112.64
1	Duration (years): intervention 6, control 4	£203.77	£267.52	£173.05	£219.75
2	Duration (years): intervention 6, control 5	£163.40	£214.39	£135.98	£171.97
3	Duration (years): intervention 4, control 4	£88.56	£116.65	£74.80	£96.08
4	Regain: Lower confidence interval	£159.52	£209.80	£134.91	£172.57
5	Regain: Upper confidence interval	£48.79	£64.22	£41.62	£53.22
6	Initial weight loss: 2.84 kg	£36.42	£47.94	£29.98	£38.09
7	Initial weight loss: 6.12 kg	£73.27	£96.07	£45.14	£55.01
	BMI of 28 or above			£96.61	£122.34

^a^Diagnosis of type 2 diabetes and prescribed single, non-insulin diabetes medication

The QALY gain per individual was 0.003 and the cost saving was £38.37. The detail of cost and QALY savings for sensitivity analysis is in Supplementary Tables 2 and 3. Per 100,000 individuals, there were 8 cases of diabetes and 23 cases of cardiovascular disease averted. For those at higher risk of diabetes (with and HbA_1c_ of between 6 and 6.5%) this increased to 49 cases of diabetes and 33 cases of CVD averted. To be cost saving, the maximum justifiable cost was £38 per-person for an intervention targeted at individuals with a high BMI and £57 per-person for those who also have an HbA_1c_ between 6 and 6.5%. It’s assumed that intervention costs are one-time costs incurred at the beginning of the intervention.

Sensitivity analysis was conducted around the duration of intervention effect, the initial weight-loss and the rate of regain. The maximum justifiable cost per person for a cost-effective intervention for the ICERs of £20,000 and £30,000 for each scenario are shown in Table 3. The largest maximum justifiable cost obtained from the sensitivity analysis was when the duration of effect was six and four years for the intervention and control group respectively and the lowest was for the lowest initial weight loss.

### Type 2 diabetes

**Fig. 3:**
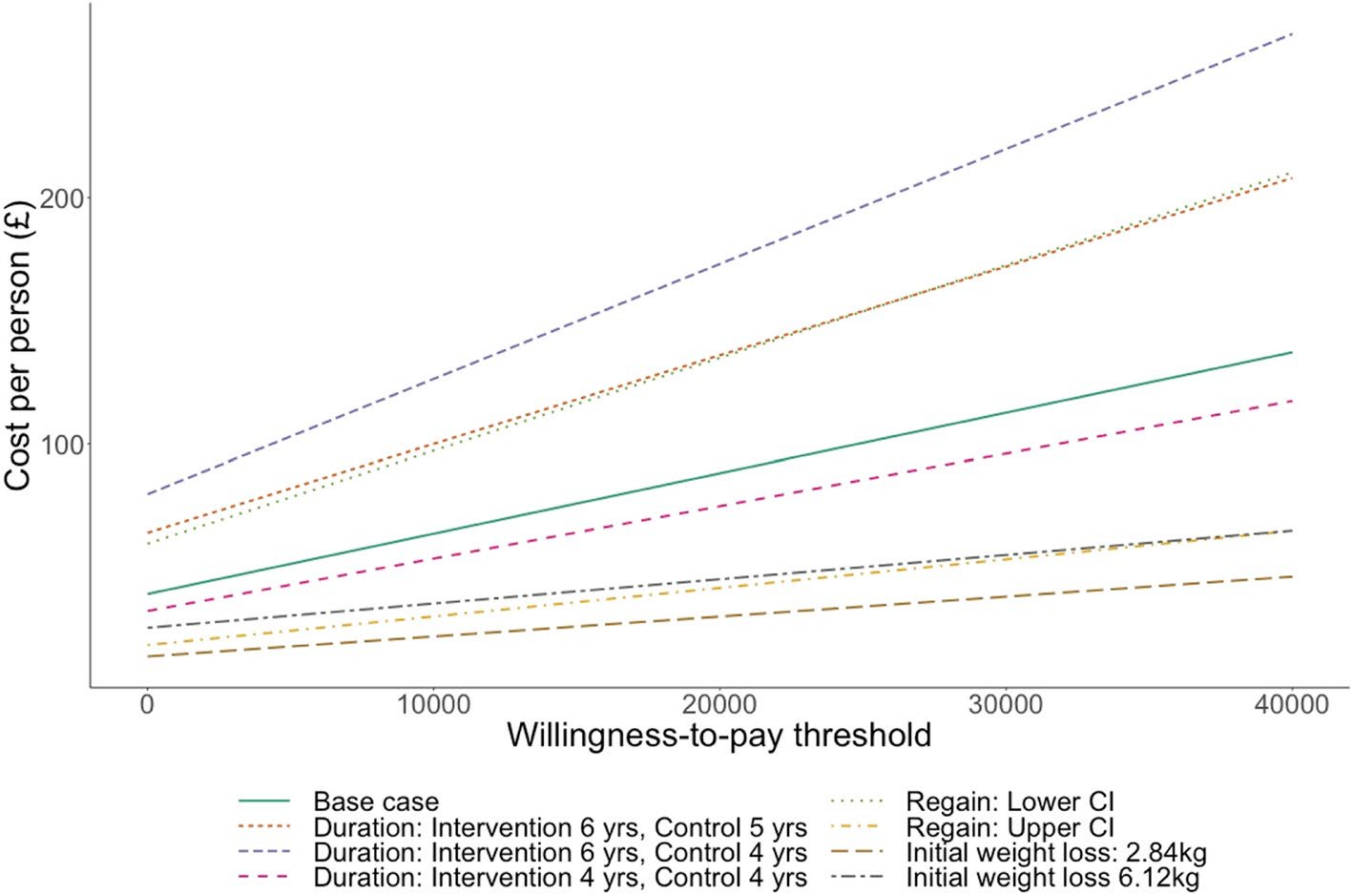
Justifiable cost per person: Base case and sensitivity analysis (Diagnosis of type 2 diabetes on single non-insulin medication)

The maximum amount that could be spent on an intervention while remaining cost-effective, with the assumption of the effect detailed in Table 1, at increasing ICER values is shown in Fig. 3. For ICERs of £20,000 and £30,000 per QALY, the maximum justifiable cost per person was £88.14 and £112.64 respectively assuming duration of effect of 5 years and health benefits accrued over the lifetime. This increased to £96.61 and £122.34 when the population was limited to individuals with a BMI of 28 or above. The QALY gain per individual was 0.002 and the cost saving was £39.14 (full details of incremental costs and QALYs for sensitivity analyses are in Additional file 1: Tables 3 and 4). There were an estimated 53 cases of CVD averted per 100,000 individuals. To be cost saving this intervention would have to cost less than £39 per-person.

Sensitivity analysis was conducted around the duration of intervention effect, the initial weight-loss and the rate of regain, and the results of this are shown for ICERS of £20,000 and £30,000 in Table 3. As found with the high BMI population, when the duration of effect was 6 years for the intervention for 4 years for the control, the maximum justifiable cost was highest, and it was lowest when the initial weight loss was 2.84 kg.

### Probability sensitivity analysis (PSA)

PSA was conducted to examine the uncertainty of the justifiable cost estimate for both groups. Supplementary Figs. 6 and 7 show the incremental cost if the justifiable cost (generated from the base case analysis) was applied to each simulation, and incremental QALYs. For both groups, over 98% of the PSA runs resulted in positive incremental QALYs. There was greater variation in incremental costs in the diabetes population; for 8.5% of PSA runs, the intervention resulted in lower costs than the control group when the mean justifiable cost is applied. For the high BMI group, when the justifiable cost is applied, over 99% of PSA runs resulted in a higher cost for the weight loss maintenance intervention compared to no intervention.

## Discussion

At an ICER of £20,000, the maximum justifiable cost was estimated to be £105 for individuals with a high BMI, £159 for individuals with a high BMI and a high HbA_1c_ (high risk of diabetes) and £88 for individuals with a diagnosis of type 2 diabetes on a single non-insulin medication. The finding that the maximum justifiable cost is lower on average for those with a diagnosis of diabetes than for those with a high BMI may seem counterintuitive given that those with a high BMI and at high risk of diabetes had the highest maximum justifiable cost. This is likely to be because, for individuals without type 2 diabetes, this intervention may be able to avert or delay a diagnosis of diabetes, which is associated with a reduction in the immediate costs associated with this diagnosis. This is particularly important for those with a high HbA_1c_ as the intervention averts or delays a potentially imminent diagnosis. Conversely, simulated individuals that have diabetes already have a higher associated cost than those without and less potential incremental gains; simulated individuals will have lower utility at the start and during the intervention period than patients with no diabetes and so the potential QALY gains are lower for patients with diabetes, and they cannot be ‘undiagnosed’ in the model. Although there is some evidence that remission from diabetes can be achieved [3] which contradicts the model assumption that type 2 diabetes is irreversible, it is not yet clear that this remission is maintained. Overall, this indicates that the benefits of intervening in high-risk individuals (and therefore preventing or delaying diabetes) are higher than the benefits of intervening in people who already have diabetes.

In sensitivity analysis, duration of effect and the initial weight loss had the greatest impact on justifiable cost. The time it takes for participants to return to their original trajectory, if they do at all, is hard to determine due to short-term follow-up within trials [4] and therefore a range of values should be considered when calculating a justifiable cost. There was a large difference between the scenario in which both the control and weight loss maintenance intervention had a duration of 4 years (£89) and the scenario in which the duration of the effect was 4 years for the control group and 6 years for the intervention group (£204) indicating the importance of the differential duration of effect between the control and intervention. The limited data on duration of weight management interventions indicates that intervention effect has diminished by an average of 5 years [4] but there is little research available on the impact of a weight maintenance intervention in the long-term and this will vary depending on the characteristics of the intervention and the control group. Researchers should consider plausible durations of effect for the control and intervention groups based on the characteristics of the planned intervention (e.g., mode of delivery or duration). The outcomes of sensitivity analysis also indicated that a weight maintenance intervention is more likely to be cost-effective for individuals with a larger initial weight loss. Previous evidence does suggest that greater initial weight-loss is associated with weight maintenance [57] supporting these findings.

Weight maintenance interventions that cost more than the maximum justifiable cost estimated are unlikely to be cost-effective based on the estimated intervention effect. While there is evidence that weight maintenance interventions are able to result in an additional 3.2 kg maintenance of weight loss over 18 months [10], there is less evidence regarding the cost. In a weight loss maintenance trial for participants that had lost at least 5% of their body weight, intervention costs were between £16 and £49 depending on the amount of face-to-face contact but it was concluded that neither intervention was likely to be cost-effective in routine practice [58]. Further evidence is required to determine the feasibility of developing an effective intervention within the justifiable costs estimated.

The method used in this analysis highlights the role that health economic modelling can have in the design and development of a new weight loss maintenance intervention. Although this type of modelling is recommended in intervention design guidance, there is little published research detailing the methods used to do this. While previous studies have used the results from a pilot trial [12], the method presented here provides an estimate of justifiable cost without a pilot trial based on a range of previous studies; this can inform the design of the trial before a pilot trial. In addition, while pre-trial modelling has been used to identify the cost of an intervention that achieves a certain risk reduction [11], the estimated impacts were not specific to a planned intervention which may limit application to certain interventions. The maximum justifiable cost provides an estimated upper bound over which the intervention would not be cost-effective, which can be compared to the predicted cost of the planned interventions. This could help to avoid an intervention which is unlikely to be cost-effective proceeding to the trial stage. Subgroup and sensitivity analysis can also inform decisions about whom the intervention should be targeted at and what factors are most likely to impact on cost-effectiveness. Although the current study is specific to a weight management intervention in the UK the methods can be applied to behavioural interventions in other health areas and countries. The increased number of public health economic models being developed [59] will facilitate this type of modelling. However, as with many public health interventions, there is likely to be a large amount of heterogeneity in effect within the patient groups and therefore there may be limited application when using the mean effect only. Additional research into the different factors that impact on the intervention effect would be informative for this type of pre-trial modelling.

There were some limitations of this analysis. Firstly, due to limited research on the impact of weight loss maintenance intervention and, in particular, the impact of weight loss maintenance interventions for people with type 2 diabetes, the same weight loss and regain was applied for each person and in both populations, despite some evidence of heterogeneity in weight trajectories [4, 57], and some differences between the baseline populations on risk factors such as systolic blood pressure. In addition, the estimate of weight regain at 24 months was based on only two intervention arms and so caution should be exercised in interpreting this result. Given the potential impact of differing weight trajectories, we conducted a range of sensitivity analysis to estimate the impact of alternate trajectories [60]. Second, individual participant data was not used for the baseline population for individuals with diabetes due to limitations on availability of data. This may limit how representative this baseline population is of a population with diabetes. Furthermore, the population was selected because they were on a single diabetes medication, but this does not rule out having been on more than one medication in the past and so the population may have been more heterogenous than the potential target population for the intervention. However, the population was generated based on many variables and based on a large dataset that is representative to the UK. Third, remission from diabetes is currently not a scenario in the model. There is some evidence that remission from diabetes (an HbA1c of below 6% and no requirement for antidiabetic medication) can be achieved by following a low-calorie diet for 3–5 months, with stepped re-introduction to food and ongoing weight loss maintenance support [3]. Given that those eligible for a weight loss maintenance intervention have already been successful in weight loss, in this study approximately 9 kg, there is a possibility that some individuals would go into remission. This means that the model may underestimate the positive impact of the intervention for those with diabetes as the cost-reduction associated with potential diabetes remission wasn’t captured. However, it is not yet clear that this remission is maintained and it’s likely that these patients will be required to attend regular screenings due to their previous diagnosis and so associated costs will still apply. Ongoing research will provide more information about the long-term impact of diabetes remission on costs and QALYs [61]. Finally, as the healthcare perspective was used, the costs incurred by patients as a result of a change in lifestyle are not considered. These costs may differentially impact subgroups, and this is not accounted for in the analysis.

## Conclusions

In conclusion, given the expected weight loss and regain estimated in the current analyses, intervention designs associated with a cost of above £105 per-person for those with a BMI of 28 or above or £88 per-person for those on first-line diabetes treatment (one medication only) should be carefully considered as these are less likely to be cost-effective. This method demonstrated, that uses results from previous relevant studies to conduct pre-trial modelling prior to a pilot study to inform the design and budgetary decisions of a weight loss maintenance intervention, can be applied to a wider range of behavioural interventions and contexts.

## Supplementary Information

**Additional file 1.** Additional tables and figures for the meta-analyses and health economic modelling.

**Additional file 2.** In-depth description of the SPHR health economic model.

**Additional file 3.** Details of parameters for SPHR health economic model.

## Data Availability

No datasets were generated or directly analysed during the current study. Restrictions apply to the Health Survey for England survey data that was used as the baseline population in the health economic model, which were used under license for use in the current study. Application to access to this data can be made through the UK data service ((https://beta.ukdataservice.ac.uk/datacatalogue/series/series?id=2000021)).

## Abbreviations

BMI: Body mass index
CVD: Cardiovascular disease
EPIC: European prospective investigation of cancer
HSE: Health survey for England
ICER: Incremental cost-effectiveness ratio
NHS: National Health Service
NICE: National institute for health and care excellence
MI: Myocardial infarction
QALY: Quality adjusted life year
SBP: Systolic blood pressure
SPHR: School of public health research
THIN: The Health Improvement Network
TIA: Trans-ischaemic attack
