# Supplementary material for: Using health economic modelling to inform the design and development of an intervention: estimating the justifiable cost of weight loss maintenance in the UK
Source: BMC Public Health. 2022 Feb 12;22:290. doi: 10.1186/s12889-022-12737-5 (PMC8840781; doi:10.1186/s12889-022-12737-5)
Supplement: Supplementary file 1 — Additional file 1. Additional tables and figures for the meta-analyses and health economic modelling. [file 12889_2022_12737_MOESM1_ESM.docx]

Additional File 1

*Table 1. Population characteristics*

| Characteristic | Population | |
| --- | --- | --- |
|  | High BMI (≥ 28kg/m2) (N=2738)^a^ | Type 2 Diabetes (N=90,219)^b^ |
| Age: mean (SD) | 53.84 | 59.8 (18.9) |
| Weight: mean (SD) | 91.33 (15.27) | 89.85 (18.96) |
| BMI: mean (SD) | 32.78 | 31.85 (6.02) |
| HbA_1c_ : mean (SD) | 5.77 (0.93) | 8.2% (2.0) |
| Systolic blood pressure: mean (SD) | 128.13 (17.10) | 137.96 (16.66) |
| Proportion with blood pressure > 160mmHg (%) | 3.42 | 10.74 |
| HDL cholesterol | 5.29 (1.08) | 5.00 (1.17) |
| Proportion with cholesterol above 5.2mmol/L | 51.36 | 38.3 |
| Gender: % male | 45.72 | 57.1 |
| Ethnicity: % White | 90.12 | 94.6 |
| BMI category: % |  |  |
| <28 | 0 | 11.33 |
| 28-34.99 | 74.99 | 60.95 |
| 35-44.99 | 20.29 | 25.17 |
| ≥45 | 2.35 | 2.55 |
| IMD^c^ quintile |  |  |
| 1 | 21.20 | 21.54 |
| 2 | 18.76 | 18.92 |
| 3 | 20.30 | 19.22 |
| 4 | 19.69 | 19.10 |
| 5 | 20.16 | 21.22 |

^a^ Population sampled from individual patient data, Health Survey for England [1]; ^b^ Population derived from summary statistics of The Health Improvement Network 2014 dataset [2];

^c^ Diagnoses of type 2 diabetes and prescribed single, non-insulin diabetes medication;

**Intervention Effect**

*Table 2. Weight loss and regain after the intervention: estimates from random-effects meta-analysis excluding those with high risk of bias)*

|  | **Treatment** | | | **Control** | | | **Difference** | | |
| --- | --- | --- | --- | --- | --- | --- | --- | --- | --- |
| **Year** | **N** | **Mean** | **95% CI** | **N** | **Mean** | **95% CI** | **N** | **Mean** | **95% CI** |
| 0 | 14 | -8.93 | (-9.49, -8.36) | 14 | -8.93 | (-9.49, -8.36) |  |  |  |
| 1 | 12 | 1.41 | (0.68, 2.14) | 12 | 2.74 | (1.73, 3.76) | 12 | -1.14 | (-2.07, -0.22) |
| 2 | 2 | 4.38 | (3.64, 5.11) | 2 | 5.6 | (5.19, 6.02) | 2 | -1.23 | (-1.96, -0.49) |

N indicates total number of intervention arms; CI: Confidence intervals; estimates are in kg

*Table 3. QALYs gained per person*

| Scenario | | At high risk of diabetes | | | Newly diagnosed with diabetes | | |
| --- | --- | --- | --- | --- | --- | --- | --- |
|  |  | Control | Active | Incremental | Control | Active | Incremental |
| **Base case** | | 11.674 | 11.678 | 0.003 | 11.882 | 11.885 | 0.002 |
| 1 | Duration (years): intervention 6, control 4 | 11.673 | 11.679 | 0.006 | 10.881 | 10.886 | 0.005 |
| 2 | Duration (years): intervention 6, control 5 | 11.674 | 11.679 | 0.005 | 10.882 | 10.886 | 0.004 |
| 3 | Duration (years): intervention 4, control 4 | 11.673 | 11.676 | 0.003 | 10.881 | 10.883 | 0.002 |
| 4 | Regain: Lower confidence interval | 11.674 | 11.679 | 0.005 | 10.882 | 10.886 | 0.004 |
| 5 | Regain: Upper confidence interval | 11.674 | 11.676 | 0.002 | 10.882 | 10.883 | 0.001 |
| 6 | Initial weight loss: 2.84kg | 11.662 | 11.663 | 0.001 | 10.872 | 10.872 | 0.001 |
| 7 | Initial weight loss: 6.12kg | 11.669 | 11.671 | 0.002 | 10.877 | 10.878 | 0.001 |

*Table 4. Costs saved gained per person*

| Scenario | | At high risk of diabetes | | | Newly diagnosed with diabetes | | |
| --- | --- | --- | --- | --- | --- | --- | --- |
|  |  | Control | Active | Incremental | Control | Active | Incremental |
| **Base case** | | 29030.10 | 28991.72 | 38.37 | 103037.93 | 102998.79 | 39.14 |
| 1 | Duration (years): intervention 6, control 4 | 29047.39 | 28971.13 | 76.26 | 103057.99 | 102978.34 | 79.65 |
| 2 | Duration (years): intervention 6, control 5 | 29030.10 | 28968.67 | 61.43 | 103037.93 | 102973.93 | 64.00 |
| 3 | Duration (years): intervention 4, control 4 | 29047.39 | 29015.01 | 32.38 | 103057.99 | 103025.74 | 32.24 |
| 4 | Regain: Lower confidence interval | 29030.10 | 28971.13 | 58.97 | 103037.93 | 102978.34 | 59.59 |
| 5 | Regain: Upper confidence interval | 29030.10 | 29012.18 | 17.91 | 103037.93 | 103019.51 | 18.42 |
| 6 | Initial weight loss: 2.84kg | 29178.22 | 29164.85 | 13.37 | 103216.06 | 103202.29 | 13.77 |
| 7 | Initial weight loss: 6.12kg | 29097.56 | 29069.90 | 27.66 | 103117.63 | 103092.22 | 25.41 |

*Figure 1. Types of interventions in studies included in a meta-analysis of weight loss maintenance studies*

*Figure 2. Forest plot from random-effects pairwise meta-analysis at 12-month post-intervention*

*Figure 3. Forest plot from random-effects pairwise meta-analysis at 24-month post-intervention*

*
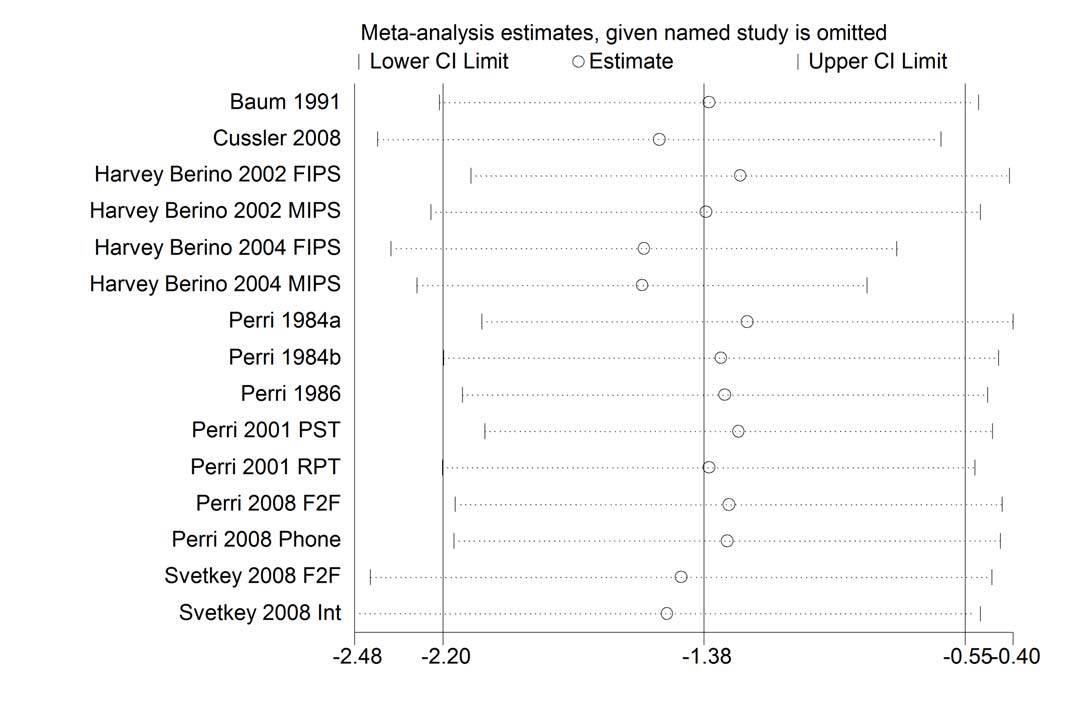
*

*Figure 4. Influence plot from random-effects pairwise meta-analysis at 12-month post-intervention*


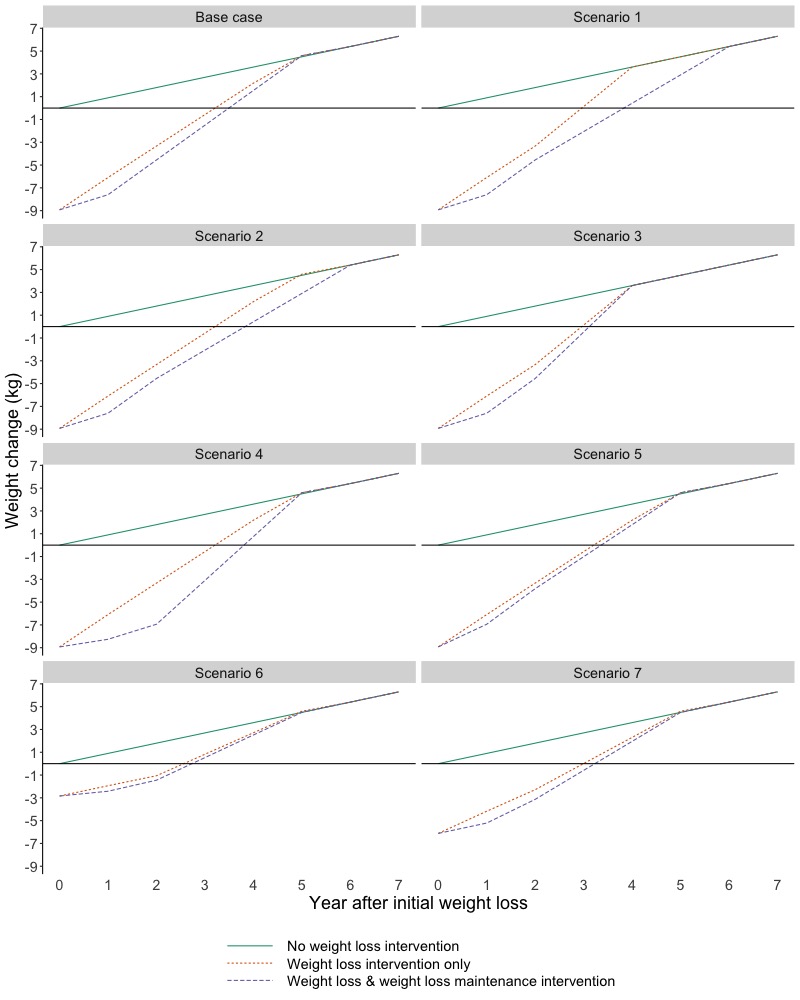


*Figure 5. Graphical representation of sensitivity analysis*

F*igure 6. Incremental cost plus justifiable cost (£104) and incremental QALYs in high BMI population (5000 Monte Carlo simulations)*

F*igure 7. Incremental cost plus justifiable cost (£88) and incremental QALYs in diabetes population (5000 Monte Carlo simulations)*

References

1. Craig R, Hirani V: **Health survey for England**. *Health and Lifestyles* 2013, **1**.
2. **The Health Improvement Network** [<https://www.the-health-improvement-network.co.uk/>]
