# Supplementary material for: Using health economic modelling to inform the design and development of an intervention: estimating the justifiable cost of weight loss maintenance in the UK
Source: BMC Public Health. 2022 Feb 12;22:290. doi: 10.1186/s12889-022-12737-5 (PMC8840781; doi:10.1186/s12889-022-12737-5)
Supplement: Supplementary file 3 — Additional file 3. Details of parameters for SPHR health economic model. [file 12889_2022_12737_MOESM3_ESM.docx]

Additional File 3: Parameters

# GP Attendance in the General Population

In the probabilistic sensitivity analysis the parameters of the South Yorkshire negative binomial model are sampled from a multivariate normal distribution, using the mean estimates described in Table 1 and covariance matrix in Table 2.

Table 1: GP attendance reported in the South Yorkshire Cohort (N= 18,437) (1)

|  | Mean | Standard error | Uncertainty Distribution |
| --- | --- | --- | --- |
| Age | 0.0076 | 0.0005 | MULTIVARIATE NORMAL |
| Male | -0.1495 | 0.0159 | MULTIVARIATE NORMAL |
| BMI | 0.0110 | 0.0015 | MULTIVARIATE NORMAL |
| Ethnicity (Non-white) | 0.2620 | 0.0375 | MULTIVARIATE NORMAL |
| Heart Disease | 0.2533 | 0.0289 | MULTIVARIATE NORMAL |
| Depression | 0.6127 | 0.0224 | MULTIVARIATE NORMAL |
| Osteoarthritis | 0.2641 | 0.0238 | MULTIVARIATE NORMAL |
| Diabetes | 0.2702 | 0.0278 | MULTIVARIATE NORMAL |
| Stroke | 0.1659 | 0.0474 | MULTIVARIATE NORMAL |
| Cancer | 0.2672 | 0.0414 | MULTIVARIATE NORMAL |
| Intercept | -0.5014 | 0.0468 | MULTIVARIATE NORMAL |
| Alpha | 0.3423 | 0.0108 | MULTIVARIATE NORMAL |

Table 2: Variance-covariance matrix for GP attendance regression

|  | Age | Male | BMI | Ethnicity (Non-white) | Heart Disease | Depression | Osteo-arthritis | Diabetes | Stroke | Cancer | Intercept | Alpha |
| --- | --- | --- | --- | --- | --- | --- | --- | --- | --- | --- | --- | --- |
| Age | 0.0000 |  |  |  |  |  |  |  |  |  |  |  |
| Male | 0.0000 | 0.0003 |  |  |  |  |  |  |  |  |  |  |
| BMI | 0.0000 | 0.0000 | 0.0000 |  |  |  |  |  |  |  |  |  |
| Ethnicity (Non-white) | 0.0000 | 0.0000 | 0.0000 | 0.0014 |  |  |  |  |  |  |  |  |
| Heart Disease | 0.0000 | 0.0000 | 0.0000 | 0.0000 | 0.0008 |  |  |  |  |  |  |  |
| Depression | 0.0000 | 0.0000 | 0.0000 | 0.0000 | 0.0000 | 0.0005 |  |  |  |  |  |  |
| Osteoarthritis | 0.0000 | 0.0000 | 0.0000 | 0.0000 | 0.0000 | 0.0000 | 0.0006 |  |  |  |  |  |
| Diabetes | 0.0000 | 0.0000 | 0.0000 | 0.0000 | -0.0001 | 0.0000 | 0.0000 | 0.0008 |  |  |  |  |
| Stroke | 0.0000 | 0.0000 | 0.0000 | 0.0000 | -0.0002 | -0.0001 | 0.0000 | -0.0001 | 0.0022 |  |  |  |
| Cancer | 0.0000 | 0.0000 | 0.0000 | 0.0000 | 0.0000 | 0.0000 | 0.0000 | 0.0000 | -0.0001 | 0.0017 |  |  |
| Intercept | 0.0000 | 0.0000 | -0.0001 | -0.0002 | 0.0002 | 0.0000 | 0.0002 | 0.0003 | 0.0000 | 0.0001 | 0.0022 |  |
| Alpha | 0.0000 | 0.0000 | 0.0000 | 0.0000 | 0.0000 | 0.0000 | 0.0000 | 0.0000 | 0.0000 | 0.0000 | 0.0000 | 0.0010 |

# Whitehall II Statistical Model of Metabolic Trajectories

The parameters derived from the Whitehall II statistical model of metabolic trajectories are described in Table 3, Table 4 and Table 5.

Table 3: Coefficient estimates for metabolic risk factor parallel growth models

|  | Parameter Description | Estimated Mean | Standard error | p-value |
| --- | --- | --- | --- | --- |
| BMI Intercept | |  |  |  |
| $\alpha_{10}$ | Population mean BMI intercept | 2.2521 | 0.045 | <0.001 |
| $\boldsymbol{\gamma}_{\boldsymbol{10}}$ | Age at baseline coefficient for BMI intercept | 0.0056 | 0.001 | <0.001 |
|  | Sex coefficient for BMI intercept | -0.0311 | 0.012 | 0.009 |
|  | Family history of CVD coefficient for BMI intercept | -0.0079 | 0.012 | 0.515 |
| $\upsilon_{10}$ | Random error term for BMI intercept | 0.1165 | 0.003 | <0.001 |
| BMI linear slope | |  |  |  |
| $\alpha_{11}$ | Population mean BMI linear slope | 0.6409 | 0.042 | <0.001 |
| $\boldsymbol{\gamma}_{\boldsymbol{11}}$ | Age at baseline coefficient for BMI linear slope | -0.0084 | 0.001 | <0.001 |
|  | Sex coefficient for BMI linear slope | -0.0285 | 0.011 | 0.009 |
|  | Family history of CVD coefficient for BMI linear slope | -0.0155 | 0.010 | 0.117 |
| $\upsilon_{11}$ | Random error term for BMI linear slope | 0.0222 | <0.001 | <0.001 |
| BMI quadratic slope | |  |  |  |
| $\alpha_{12}$ | Population mean BMI quadratic slope | -0.2007 | 0.023 | <0.001 |
| $\boldsymbol{\gamma}_{\boldsymbol{12}}$ | Age at baseline coefficient for quadratic slope | 0.0026 | <0.001 | <0.001 |
|  | Sex coefficient for quadratic slope | 0.0089 | 0.006 | 0.147 |
|  | Family history of CVD coefficient for quadratic slope | 0.0104 | 0.006 | 0.061 |
| $\varepsilon_{1}$ | Random error term for BMI | 0.0104 | <0.001 | <0.001 |
| Glyc Intercept | |  |  |  |
| $\alpha_{20}$ | Population mean glyc intercept | 0 | NA | NA |
| $\boldsymbol{\gamma}_{\boldsymbol{20}}$ | Smoker coefficient for glyc intercept | -0.1388 | 0.029 | <0.001 |
| $\tau_{20}$ | Association between BMI intercept and glyc intercept | 0.2620 | 0.024 | <0.001 |
| $\upsilon_{20}$ | Random error term for glyc intercept | 0.0851 | 0.008 | <0.001 |
| Glyc linear slope | |  |  |  |
| $\alpha_{21}$ | Population mean glyc linear slope | -0.4255 | 0.071 | <0.001 |
| $\boldsymbol{\gamma}_{\boldsymbol{21}}$ | Sex coefficient for glyc linear slope | 0.1486 | 0.045 | 0.001 |
|  | Ethnicity coefficient for glyc linear slope | -0.0218 | 0.081 | 0.786 |
|  | Family history of T2DM coefficient for glyc linear slope | -0.0512 | 0.054 | 0.345 |
|  | Smoker coefficient for glyc linear slope | 0.1796 | 0.066 | 0.007 |
| $\tau_{21}$ | Association between BMI intercept and glyc linear slope | 0.0821 | 0.024 | 0.001 |
| $\tau_{22}$ | Association between BMI linear slope and glyc linear slope | 0.1984 | 0.073 | 0.007 |
| $\upsilon_{21}$ | Random error term for glyc linear slope | 0.0222 | 0.011 | 0.053 |
| Glyc quadratic slope | |  |  |  |
| $\alpha_{22}$ | Population mean glyc quadratic slope | 0.1094 | 0.025 | <0.001 |
| $\boldsymbol{\gamma}_{\boldsymbol{22}}$ | Sex coefficient for glyc quadratic slope | -0.0855 | 0.027 | 0.002 |
|  | Ethnicity coefficient for glyc quadratic slope | 0.0899 | 0.049 | 0.067 |
|  | Family history of T2DM coefficient for glyc quadratic slope | 0.0633 | 0.033 | 0.052 |
|  | Smoker coefficient for glyc quadratic slope | -0.0390 | 0.040 | 0.330 |
| $\upsilon_{22}$ | Random error term for glyc quadratic slope | 0.0107 | 0.003 | 0.002 |
| $\varepsilon_{2}$ | Glyc measurement error | 0.0707 | 0.005 | <0.001 |
| SBP Intercept | |  |  |  |
| $\alpha_{30}$ | Population mean SBP intercept | 0.6934 | 0.021 | <0.001 |
| $\boldsymbol{\gamma}_{\boldsymbol{30}}$ | Age at baseline coefficient for SBP intercept | 0.0043 | <0.001 | <0.001 |
|  | Sex coefficient for SBP intercept | 0.0380 | 0.004 | <0.001 |
|  | Smoking coefficient for SBP intercept | -0.0243 | 0.006 | <0.001 |
|  | Ethnicity coefficient for SBP intercept | 0.0078 | 0.007 | 0.300 |
|  | Family history of CVD coefficient for SBP intercept | 0.0061 | 0.004 | 0.160 |
| $\boldsymbol{\tau}_{\boldsymbol{31}}$ | Association between BMI intercept and SBP intercept | 0.1080 | 0.006 | <0.001 |
| $\upsilon_{30}$ | Random error term for SBP intercept | 0.0085 | 0.00 | <0.001 |
| SBP linear slope | |  |  |  |
| $\alpha_{31}$ | Population mean SBP linear slope | -0.0227 | 0.021 | 0.278 |
| $\boldsymbol{\gamma}_{\boldsymbol{31}}$ | Age at baseline coefficient for SBP linear slope | 0.0024 | <0.001 | <0.001 |
|  | Sex coefficient for SBP linear slope | -0.0004 | 0.004 | 0.927 |
|  | Smoking coefficient for SBP linear slope | 0.0205 | 0.005 | <0.001 |
|  | Ethnicity coefficient for SBP linear slope | 0.0224 | 0.007 | 0.001 |
|  | Family history of CVD coefficient for SBP linear slope | -0.0013 | 0.004 | 0.748 |
| $\boldsymbol{\tau}_{\boldsymbol{31}}$ | Association between BMI intercept and SBP linear slope | -0.0396 | 0.006 | <0.001 |
|  | Association between BMI linear slope and SBP linear slope | 0.2325 | 0.019 | <0.001 |
| $\upsilon_{31}$ | Random error term for SBP linear slope | 0.0024 | <0.001 | <0.001 |
| $\varepsilon_{3}$ | SBP measurement error variance | 0.0093 | <0.001 | <0.001 |
| TC Intercept | |  |  |  |
| $\alpha_{40}$ | Population mean TC intercept | 2.9956 | 0.176 | <0.001 |
| $\boldsymbol{\gamma}_{\boldsymbol{40}}$ | Age at baseline coefficient for TC intercept | 0.0456 | 0.003 | <0.001 |
|  | Sex coefficient for TC intercept | 0.0660 | 0.036 | 0.070 |
| $\tau_{40}$ | Association between BMI intercept and TC intercept | 0.4459 | 0.049 | <0.001 |
| $\upsilon_{40}$ | Random error term for TC intercept | 0.8960 | 0.025 | <0.001 |
| TC linear slope | |  |  |  |
| $\alpha_{41}$ | Population mean TC linear slope | 2.1216 | 0.128 | <0.001 |
| $\boldsymbol{\gamma}_{\boldsymbol{41}}$ | Age at baseline coefficient for TC linear slope | -0.0316 | 0.002 | <0.001 |
|  | Sex coefficient for TC linear slope | -0.2677 | 0.026 | <0.001 |
| $\tau_{41}$ | Association between BMI intercept and TC linear slope | -0.4808 | 0.035 | <0.001 |
| $\tau_{42}$ | Association between BMI linear slope and TC linear slope | 0.9802 | 0.108 | <0.001 |
| $\upsilon_{41}$ | Random error term for TC linear slope | 0.1583 | 0.011 | <0.001 |
| $\varepsilon_{4}$ | TC measurement error variance | 0.3426 | 0.006 | <0.001 |
| HDL Intercept | |  |  |  |
| $\alpha_{50}$ | Population mean HDL intercept | 2.4124 | 0.054 | <0.001 |
| $\boldsymbol{\gamma}_{\boldsymbol{50}}$ | Age at baseline coefficient for HDL intercept | 0.0032 | 0.011 | <0.001 |
|  | Sex coefficient for HDL intercept | -0.3710 | 0.001 | <0.001 |
| $\tau_{51}$ | Association between BMI intercept and HDL intercept | -0.3514 | 0.015 | <0.001 |
| $\upsilon_{50}$ | Random error term for HDL intercept | 0.0827 | -0.040 | <0.001 |
| HDL linear slope | |  |  |  |
| $\alpha_{51}$ | Population mean HDL linear slope | 0.1241 | 0.034 | <0.001 |
| $\boldsymbol{\gamma}_{\boldsymbol{51}}$ | Age at baseline coefficient for HDL linear slope | 0.0020 | 0.001 | <0.001 |
|  | Sex coefficient for HDL linear slope | 0.0041 | 0.007 | 0.558 |
| $\boldsymbol{\tau}_{\boldsymbol{51}}$ | Association between BMI intercept and HDL linear slope | -0.0400 | 0.010 | <0.001 |
| $\upsilon_{51}$ | Random error term for HDL linear slope | 0.0090 | 0.001 | <0.001 |
| $\varepsilon_{5}$ | HDL measurement error variance | 0.0333 | 0.001 | <0.001 |

Table 4: Coefficient estimates for latent glycaemic measurement model

|  | Parameter Description | Estimated Mean | Standard error | p-value |
| --- | --- | --- | --- | --- |
| $\mu_{0}$ | FPG intercept | 4.2903 | 0.089 | <0.001 |
| $\theta_{01}$ | Glycaemic factor to FPG | 1 | NA | NA |
| $\theta_{02}$ | Age to FPG | 0.0031 | 0.001 | 0.022 |
| $\theta_{03}$ | Sex to FPG | 0.2129 | 0.021 | <0.001 |
| $\theta_{04}$ | Ethnicity to FPG | 0.0100 | 0.037 | 0.786 |
| $\theta_{05}$ | Family history of diabetes to FPG | 0.1168 | 0.025 | <0.001 |
| $\varepsilon_{0}$ | FPG measurement error variance | 0.1649 | 0.007 | <0.001 |
| $\mu_{1}$ | 2-hr Glucose intercept | 0.5707 | 0.223 | 0.011 |
| $\theta_{11}$ | Glycaemic factor to 2-hr glucose | 2.4384 | 0.078 | <0.001 |
| $\theta_{12}$ | Age to 2-hr glucose | 0.0716 | 0.003 | <0.001 |
| $\theta_{13}$ | Sex to 2-hr glucose | -0.1411 | 0.058 | 0.014 |
| $\theta_{14}$ | Ethnicity to 2-hr glucose | 0.3047 | 0.100 | 0.002 |
| $\theta_{15}$ | Family history of diabetes to 2-hr glucose | 0.3496 | 0.068 | <0.001 |
| $\varepsilon_{1}$ | 2-hr measurement error variance | 2.3679 | 0.054 | <0.001 |
| $\mu_{2}$ | HbA1c intercept | 4.4769 | 0.073 | <0.001 |
| $\theta_{21}$ | Glycaemic factor to HBA1c | 0.5074 | 0.016 | <0.001 |
| $\theta_{22}$ | Age to HBA1c | 0.0101 | 0.001 | <0.001 |
| $\theta_{23}$ | Sex to HBA1c | -0.0457 | 0.001 | <0.001 |
| $\theta_{24}$ | Ethnicity to HBA1c | 0.1854 | 0.030 | <0.001 |
| $\theta_{25}$ | Family history of diabetes to HBA1c | 0.0563 | 0.020 | 0.004 |
| $\varepsilon_{2}$ | HbA1c measurement error variance | 0.1166 | 0.003 | <0.001 |

Table 5: Covariance matrix $\boldsymbol{\Omega}$ for individual random error

|  | $\upsilon_{10}$ | $\upsilon_{11}$ | $\upsilon_{20}$ | $\upsilon_{21}$ | $\upsilon_{22}$ | $\upsilon_{30}$ | $\upsilon_{31}$ | $\upsilon_{40}$ | $\upsilon_{41}$ | $\upsilon_{50}$ | $\upsilon_{51}$ |
| --- | --- | --- | --- | --- | --- | --- | --- | --- | --- | --- | --- |
| $\upsilon_{10}$ | 0.1165 |  |  |  |  |  |  |  |  |  |  |
| $\upsilon_{11}$ | 0.0095 | 0.0131 |  |  |  |  |  |  |  |  |  |
| $\upsilon_{20}$ | <0.0010 | <0.0010 | 0.0851 |  |  |  |  |  |  |  |  |
| $\upsilon_{21}$ | <0.0010 | <0.0010 | 0.0222 | 0.0209 |  |  |  |  |  |  |  |
| $\upsilon_{22}$ | <0.0010 | <0.0010 | <0.0010 | <0.0010 | 0.0107 |  |  |  |  |  |  |
| $\upsilon_{30}$ | <0.0010 | <0.0010 | 0.0080 | <0.0010 | <0.0010 | 0.0085 |  |  |  |  |  |
| $\upsilon_{31}$ | <0.0010 | <0.0010 | <0.0010 | 0.0018 | <0.0010 | <0.0017 | 0.0024 |  |  |  |  |
| $\upsilon_{40}$ | <0.0010 | <0.0010 | 0.0324 | <0.0010 | <0.0010 | 0.0031 | <0.0010 | 0.8960 |  |  |  |
| $\upsilon_{41}$ | <0.0010 | <0.0010 | <0.0010 | -<0.0012 | <0.0010 | <0.0010 | 0.0066 | -0.2229 | 0.1583 |  |  |
| $\upsilon_{50}$ | <0.0010 | <0.0010 | -0.0118 | <0.0010 | <0.0010 | 0.0010 | <0.0010 | 0.0273 | <0.0010 | 0.0827 |  |
| $\upsilon_{51}$ | <0.0010 | <0.0010 | <0.0010 | -0.0059 | <0.0010 | <0.0010 | 0.0020 | <0.0010 | 0.0159 | 0.0061 | 0.0090 |

## HbA1c trajectory in individuals diagnosed with type 2 diabetes

The input parameters for the initial reduction in HbA1c and long term trend in HbA1c following diagnosis, derived from analysis of the UKPDS outcomes model (2), are reported in Table 6 and Table 7 respectively.

Table 6: Estimated change in HbA1c in first year following diabetes diagnosis

|  | Distribution | Parameter 1 | Parameter 2 | Central estimate |
| --- | --- | --- | --- | --- |
| Change in HbA1c Intercept | NORMAL | -2.9465 | 0.0444513 | -2.9465 |
| HbA1c at baseline | NORMAL | 0.5184 | 0.4521958 | 0.5184 |

Table 7: Estimated change in HbA1c following diabetes diagnosis over long term

| Parameter Description | Distribution | Parameter 1 | Parameter 2 | Central estimate |
| --- | --- | --- | --- | --- |
| Longitudinal HbA1c for diabetes intercept | NORMAL | -0.024 | 0.017 | -0.024 |
| Longitudinal HbA1c for diabetes log(time since diagnosis) | NORMAL | 0.144 | 0.009 | 0.144 |
| Longitudinal HbA1c for diabetes Second year | NORMAL | -0.333 | 0.05 | -0.333 |
| Longitudinal HbA1c for diabetes lag HbA1c | NORMAL | 0.759 | 0.004 | 0.759 |
| Longitudinal HbA1c for diabetes HbA1c at diagnosis | NORMAL | 0.085 | 0.004 | 0.0896 |

## Systolic blood pressure and cholesterol trajectory following treatment

The changes in systolic blood pressure and total cholesterol following treatment with anti-hypertensives or statins and statin uptake are reported in Table 8.

Table 8: Treatment effects following treatment

| Parameter Description | Distribution | Parameter 1 | Parameter 2 | Central estimate | Source |
| --- | --- | --- | --- | --- | --- |
| Simvastatin treatment effects | NORMAL | -1.45 | 0.11 | -1.45 | (3) |
| Anti-hypertensive treatment effect | NORMAL | -8.4 | 0.638 | -8.4 | (4) |
| Statin Uptake | UNIFORM | 0.65 | (0.4-0.9) | 0.65 | (5) |

## Metabolic Risk Factor screening

The distribution for the HbA1c threshold at which opportunistic screening for type 2 Diabetes is initiated even if the individual does not have a history of cardiovascular disease, microvascular disease or identified impaired glucose regulation is reported in Table 9.

Table 9: Threshold for HbA1c opportunistic diagnosis

| Parameter Description | Distribution | Parameter 1 | Parameter 2 | Central estimate | Source |
| --- | --- | --- | --- | --- | --- |
| HbA1c at diagnosis | NORMAL | 8.1 | 0.073 | 8.1 | (6) |

# Comorbid Outcomes and Mortality

## Cardiovascular disease

The parameter distributions for men and women based on the QRISK2 model (7) are reported in Table 10.

Table 10: Input parameters of the QRISK2 risk model

| Parameter Description | Distribution | Parameter 1 | Parameter 2 | Central estimate |
| --- | --- | --- | --- | --- |
| QRISK female ethnicity 2 | NORMAL | 0.2163 | 0.0537 | 0.2163 |
| QRISK female ethnicity 3 | NORMAL | 0.6905 | 0.069 | 0.6905 |
| QRISK female ethnicity 4 | NORMAL | 0.3423 | 0.1073 | 0.3423 |
| QRISK female ethnicity 5 | NORMAL | 0.0731 | 0.1071 | 0.0731 |
| QRISK female ethnicity 6 | NORMAL | -0.0989 | 0.0619 | -0.0989 |
| QRISK female ethnicity 7 | NORMAL | -0.2352 | 0.1275 | -0.2352 |
| QRISK female ethnicity 8 | NORMAL | -0.2956 | 0.1721 | -0.2956 |
| QRISK female ethnicity 9 | NORMAL | -0.1010 | 0.0793 | -0.1010 |
| QRISK female smoke 2 | NORMAL | 0.2033 | 0.0152 | 0.2033 |
| QRISK female smoke 3 | NORMAL | 0.48200 | 0.0220 | 0.4820 |
| QRISK female smoke 4 | NORMAL | 0.6126 | 0.0178 | 0.6126 |
| QRISK female smoke 5 | NORMAL | 0.7481 | 0.0194 | 0.7481 |
| QRISK female age 1 | NORMAL | 5.0373 | 1.0065 | 5.0327 |
| QRISK female age 2 | NORMAL | -0.0108 | 0.0022 | -0.0108 |
| QRISK female bmi | NORMAL | 0.4724 | 0.0423 | 0.4724 |
| QRISK female cholesterol | NORMAL | 0.6375 | 0.0143 | 0.6375 |
| QRISK female sbp | NORMAL | 0.0106 | 0.0045 | 0.0106 |
| QRISK female townsend | NORMAL | 0.060 | 0.0068 | 0.060 |
| QRISK female fibrillation | NORMAL | 1.3261 | 0.0310 | 1.3261 |
| QRISK female RA | NORMAL | 0.3626 | 0.0319 | 0.3626 |
| QRISK female Renal | NORMAL | 0.7636 | 0.0639 | 0.7636 |
| QRISK female Hypertension | NORMAL | 0.5421 | 0.0115 | 0.5421 |
| QRISK female diabetes | NORMAL | 0.8940 | 0.0199 | 0.8940 |
| QRISK female family history cvd | NORMAL | 0.5997 | 0.0122 | 0.5997 |
| QRISK female age1 * smoke 1 | NORMAL | 0.1774 | 0.0355 | 0.1774 |
| QRISK female age 1 * smoke 2 | NORMAL | -0.3277 | 0.0655 | -0.3277 |
| QRISK age1 * smoke 3 | NORMAL | -1.1533 | 0.2307 | -1.1533 |
| QRISK female age 1 * smoke 4 | NORMAL | -1.5397 | 0.3079 | -1.5397 |
| QRISK female age 1 * atrial fibrillation | NORMAL | -4.6084 | 0.922 | -4.6084 |
| QRISK female age 1 * renal | NORMAL | -2.6401 | 0.5280 | -2.6401 |
| QRISK female age 1 * hypertension | NORMAL | -2.2480 | 0.4496 | -2.2480 |
| QRISK female age 1 * diabetes | NORMAL | -1.8452 | 0.3690 | -1.8452 |
| QRISK female age 1 * bmi | NORMAL | -3.0851 | 0.6170 | -3.0851 |
| QRISK female age 1 * family history cvd | NORMAL | -0.2481 | 0.0496 | -0.2481 |
| QRISK female age 1 * sbp | NORMAL | -0.0132 | 0.0026 | -0.0132 |
| QRISK female age 1 * town | NORMAL | -0.0369 | 0.0074 | -0.0369 |
| QRISK female age 2 * smoke 1 | NORMAL | -0.0053 | 0..0001 | -0.0053 |
| QRISK female age 2 * smoke 2 | NORMAL | -0.0005 | 0.0001 | -0.0005 |
| QRISK female age 2 * smoke 3 | NORMAL | -0.0105 | 0.0021 | -0.0105 |
| QRISK female age 2 * smoke 4 | NORMAL | -0.0155 | 0.0031 | -0.0155 |
| QRISK female age 2 * fibrillation | NORMAL | -0.0507 | 0.0101 | -0.0507 |
| QRISK female age 2 * renal | NORMAL | 0.0343 | 0.0069 | 0.0343 |
| QRISK female age 2 * hypertension | NORMAL | 0.0258 | 0.0051 | 0.0258 |
| QRISK female age 2 * diabetes | NORMAL | 0.0180 | 0.0036 | 0.0180 |
| QRISK female age 2 * bmi | NORMAL | 0.0345 | 0.0069 | 0.0345 |
| QRISK female age 2 * family history cardiovascular | NORMAL | -0.0062 | 0.0012 | -0.0062 |
| QRISK female age 2 * sbp | NORMAL | -0.000029 | 0.000006 | -0.000029 |
| QRISK female age 2 * townsend | NORMAL | -0.0011 | 0.0002 | -0.0011 |
| QRISK female 1 year survival | CONSTANT | 0.9983 | NA | NA |
| QRISK male ethnicity 2 | NORMAL | 0.3163 | 0.0425 | 0.3163 |
| QRISK male ethnicity 3 | NORMAL | 0.6092 | 0.0547 | 0.6092 |
| QRISK male ethnicity 4 | NORMAL | 0.5958 | 0.0727 | 0.5958 |
| QRISK male ethnicity 5 | NORMAL | 0.1142 | 0.0845 | 0.1142 |
| QRISK male ethnicity 6 | NORMAL | -0.3489 | 0.0641 | -0.3489 |
| QRISK male ethnicity 7 | NORMAL | -0.3604 | 0.1094 | -0.3604 |
| QRISK male ethnicity 8 | NORMAL | -0.2666 | 0.1538 | -0.2666 |
| QRISK male ethnicity 9 | NORMAL | -0.1208 | 0.0734 | -0.1208 |
| QRISK male SMOKE 2 | NORMAL | 0.2033 | 0.0152 | 0.2033 |
| QRISK male SMOKE 3 | NORMAL | 0.4820 | 0.0220 | 0.4820 |
| QRISK male SMOKE 4 | NORMAL | 0.6126 | 0.0178 | 0.6126 |
| QRISK male SMOKE 5 | NORMAL | 0.7481 | 0.0194 | 0.7481 |
| QRISK male age 1 | NORMAL | 47.316 | 9..4630 | 47.316 |
| QRISK male age 2 | NORMAL | -101.236 | 20.247 | -101.236 |
| QRISK male bmi | NORMAL | 0.5425 | 0.0299 | 0.5425 |
| QRISK male cholesterol | NORMAL | 0.14425 | 0.0022 | 0.14425 |
| QRISK male sbp | NORMAL | 0.0081 | 0.0046 | 0.0081 |
| QRISK male townsend | NORMAL | 0.0365 | 0.0048 | 0.0365 |
| QRISK male fibrillation | NORMAL | 0.7547 | 0.1018 | 0.7547 |
| QRISK male RA | NORMAL | 0.3089 | 0.0445 | 0.3089 |
| QRISK male renal | NORMAL | 0.7441 | 0.0702 | 0.7441 |
| QRISK male hypertension | NORMAL | 0.6965 | 0.011 | 0.6965 |
| QRISK male age 1 smoke 1 | NORMAL | -3.8805 | 0.7761 | -3.8805 |
| QRISK male age 1 smoke 2 | NORMAL | -16.703 | 3.3406 | -16.703 |
| QRISK male age 1 smoke 3 | NORMAL | -15.3738 | 3.5291 | -15.3738 |
| QRISK male age 1 smoke 4 | NORMAL | -17.6453 | 3.5291 | -17.6453 |
| QRISK male age 1 fibrillation | NORMAL | -7.0146 | 1.4056 | -7.0282 |
| QRISK male age 1 renal | NORMAL | -17.015 | 3.4029 | -17.015 |
| QRISK male age 1 hypertension | NORMAL | 33.9625 | 6.7925 | 33.9625 |
| QRISK male age 1 diabetes | NORMAL | 12.7886 | 2.5577 | 12.7886 |
| QRISK male age 1 bmi | NORMAL | 3.2680 | 0.6536 | 3.2680 |
| QRISK male age 1 fxcd | NORMAL | -17.9219 | 3.5844 | -17.9219 |
| QRISK male age 1 sbp | NORMAL | -0.1511 | 0.030 | -0.1511 |
| QRISK male age 1 town | NORMAL | -2.5502 | 0.5100 | -2.5502 |
| QRISK male age 2 SMOKE 1 | NORMAL | 7.9709 | 1.5942 | 7.9709 |
| QRISK male age 2 SMOKE 2 | NORMAL | 23.6859 | 4.7372 | 23.6859 |
| QRISK male age 2 SMOKE 3 | NORMAL | 23.1371 | 4.6274 | 23.1371 |
| QRISK male age 2 SMOKE 4 | NORMAL | 26.8674 | 5.3735 | 26.8674 |
| QRISK male age 2 Fibrillation | NORMAL | 14.4518 | 2.8904 | 14.4518 |
| QRISK male age 2 renal | NORMAL | 28.2702 | 5.654 | 28.2702 |
| QRISK male age 2 hypertension | NORMAL | -18.8167 | 3.7633 | -18.8167 |
| QRISK male age 2 diabetes | NORMAL | 0.9630 | 0.1926 | 0.963 |
| QRISK male age 2 bmi | NORMAL | 10.5517 | 2.1103 | 10.5517 |
| QRISK male age 2 FXCD | NORMAL | 26.6047 | 5.3209 | 26.6047 |
| QRISK male age 2 sbp | NORMAL | 0.2911 | 0.0582 | 0.2911 |
| QRISK male age 2 town | NORMAL | 3.007 | 0.6014 | 3.007 |
| QRISK2 male 1 year survival | CONSTANT | 0.997 | NA | NA |

The QRISK2 model was modified to allow a linear relationship between HbA1c and the risk of cardiovascular disease for individuals with Impaired Glucose tolerance and type 2 Diabetes (HbA1c>42 mmol/mol). The parameter distributions for these additional inputs are reported in Table 11.

Table 11: Additional parameters for linear relationship between HbA1c and cardiovascular disease

| Parameter Description | Distribution | Parameter 1 | Parameter 2 | Central estimate | Source |
| --- | --- | --- | --- | --- | --- |
| Female RR of MI due to HbA1c in diabetics | LOGNORMAL | 0.078 | 0.030 | 1.08 | (8) |
| Male RR of MI due to HbA1c in diabetics | LOGNORMAL | 0.108 | 0.023 | 1.11 | (8) |
| RR of stroke due to HbA1c in diabetics | LOGNORMAL | 0.092 | 0.026 | 1.096 | (8) |
| Log(RR) of cvd due to IGR | NORMAL | 0.223 | 0.043 | 1.25 | (9) |

## Congestive Heart Failure

The parameter distributions for congestive heart failure based on the Framingham Heart Study (10) are reported in Table 12.

Table 12: Input parameters for Congestive Heart Failure Risk model for men and women

| Parameter Description | Distribution | Parameter 1 | Parameter 2 | Central estimate |
| --- | --- | --- | --- | --- |
| Male Heart failure baseline hazard | NORMAL | -9.2087 | 0.9209 | -9.2087 |
| Male Heart failure Age | NORMAL | 0.0412 | 0.0278 | 0.0412 |
| Male Heart failure LVH | NORMAL | 0.9026 | 1.0359 | 0.9026 |
| Male Heart failure Heart rate | NORMAL | 0.0166 | 0.0174 | 0.0166 |
| Male Heart failure Systolic blood pressure | NORMAL | 0.00804 | 0.0117 | 0.00804 |
| Male Heart failure CHD | NORMAL | 1.6079 | 0.5336 | 1.6079 |
| Male Heart failure Valve disease | NORMAL | 0.9714 | 0.6557 | 0.9714 |
| Male Heart failure Diabetes | NORMAL | 0.2244 | 0.6682 | 0.2244 |
| Female Heart failure baseline hazard | NORMAL | -10.7988 | 1.0799 | -10.7988 |
| Female Heart failure Age | NORMAL | 0.0503 | 0.0301 | 0.0503 |
| Female Heart failure LVH | NORMAL | 1.3402 | 0.8298 | 1.3402 |
| Female Heart failure Heart rate | NORMAL | 0.0105 | 0.0193 | 0.0105 |
| Female Heart failure Systolic blood pressure | NORMAL | 0.00337 | 0.0109 | 0.00337 |
| Female Heart failure CHD | NORMAL | 1.5549 | 0.5973 | 1.5549 |
| Female Heart failure Valve disease | NORMAL | 1.3929 | 0.6707 | 1.3929 |
| Female Heart failure Diabetes | NORMAL | 1.3857 | 0.7105 | 1.3857 |
| Female Heart failure BMI | NORMAL | 0.0578 | 0.0555 | 0.0578 |
| Female Heart failure Valve disease | NORMAL | -0.986 | 1.4370 | -0.986 |

## Microvascular Complications

The parameter distributions for the risk models for foot ulcer, blindness, renal failure, first amputation and second amputation are reported in Table 13. Parameters for renal failure were based on the UKPDS Outcomes Model 1 (2), whereas parameters for other microvascular complications were based on the UKPDS Outcomes Model 2 (8).

Table 13: Input parameters for microvascular complications

| Parameter Description | Distribution | Parameter 1 | Parameter 2 | Central estimate |
| --- | --- | --- | --- | --- |
| Renal failure baseline hazard | NORMAL | -10.016 | 0.939 | -10.016 |
| Renal failure Weibull shape | NORMAL | 1.865 | 1.4352 | 1.865 |
| Renal failure systolic blood pressure | NORMAL | 0.404 | 0.106 | 0.404 |
| Renal failure blindness | NORMAL | 2.082 | 0.551 | 2.082 |
| Foot ulcer baseline hazard | NORMAL | -11.295 | 1.13 | -11.295 |
| Foot ulcer age at diagnosis | NORMAL | 0.043 | 0.014 | 0.043 |
| Foot ulcer female | NORMAL | -0.962 | 0.255 | -0.962 |
| Foot ulcer BMI | NORMAL | 0.053 | 0.019 | 0.053 |
| Foot ulcer HbA1c | NORMAL | 0.16 | 0.056 | 0.16 |
| Foot ulcer PVD | NORMAL | 0.968 | 0.258 | 0.968 |
| Amputation baseline hazard | NORMAL | -14.844 | 1.205 | -14.844 |
| Amputation age at diagnosis | NORMAL | 0.023 | 0.011 | 0.023 |
| Amputation female | NORMAL | -0.445 | 0.189 | -0.445 |
| Amputation atrial fibrillation | NORMAL | 1.088 | 0.398 | 1.088 |
| Amputation HbA1c | NORMAL | 0.248 | 0.042 | 0.248 |
| Amputation HDL | NORMAL | -0.059 | 0.032 | -0.059 |
| Amputation heart rate | NORMAL | 0.098 | 0.05 | 0.098 |
| Amputation MMALB | NORMAL | 0.602 | 0.18 | 0.602 |
| Amputation peripheral vascular disease | NORMAL | 1.01 | 0.189 | 1.01 |
| Amputation white blood count | NORMAL | 0.04 | 0.017 | 0.04 |
| Amputation Stroke | NORMAL | 1.299 | 0.245 | 1.299 |
| Amputation shape | NORMAL | 2.067 | 0.193 | 2.067 |
| Amputation with Ulcer lambda | NORMAL | -0.881 | 0139 | -0.881 |
| Amputation with Ulcer age at diagnosis | NORMAL | -0.065 | 0.027 | -0.065 |
| Amputation with Ulcer PVD | NORMAL | 1.769 | 0.449 | 1.769 |
| Second Amputation baseline hazard | NORMAL | -3.455 | 0.565 | -3.455 |
| Second Amputation HbA1c | NORMAL | 0.127 | 0.06 | 0.127 |
| Blindness baseline hazard | NORMAL | -10.6774 | 0.759 | -10.6774 |
| Blindness age at diagnosis | NORMAL | 0.047 | 0.009 | 0.047 |
| Blindness HbA1c | NORMAL | 0.171 | 0.032 | 0.171 |
| Blindness heart rate | NORMAL | 0.08 | 0.039 | 0.08 |
| Blindness systolic blood pressure | NORMAL | 0.068 | 0.032 | 0.068 |
| Blindness white blood cells | NORMAL | 0.052 | 0.019 | 0.052 |
| Blindness CHF | NORMAL | 0.841 | 0.287 | 0.841 |
| Blindness IHD | NORMAL | 0.61 | 0.208 | 0.61 |

## Cancer

The parameter distributions for the incidence and hazard ratios for breast cancer and colorectal cancer are reported in Table 14.

Table 14: Input parameters for breast cancer and colorectal cancer risk models

| Parameter Description | Distribution | Parameter 1 | Parameter 2 | Central estimate | Source |
| --- | --- | --- | --- | --- | --- |
| Colorectal cancer men | NORMAL | 0.0011 | 0.0001 | 0.0011 | (11) |
| Colorectal cancer women | NORMAL | 0.0005 | 0.0000 | 0.0005 | (11) |
| Breast cancer pre-menopause | NORMAL | 0.0010 | 0.0001 | 0.0010 | (12) |
| Breast cancer post-menopause | NORMAL | 0.0028 | 0.0002 | 0.0028 | (12) |
| Colorectal cancer BMI relative risk for men | LOGNORMAL | 0.1906 | 0.0111 | 1.21 | (13) |
| Colorectal cancer BMI relative risk for women | LOGNORMAL | 0.0392 | 0.0151 | 1.04 | (13) |
| Breast cancer BMI relative risk for pre-menopause | LOGNORMAL | -0.1165 | 0.0251 | 0.89 | (13) |
| Breast cancer BMI relative risk for post-menopause | LOGNORMAL | 0.0862 | 0.0205 | 1.09 | (13) |

The parameter distributions for breast and colorectal cancer mortality are reported in Table 15.

Table 15: Input parameters for breast cancer and colorectal cancer mortality (14)

| Parameter Description | Distribution | Parameter 1 | Parameter 2 | Central estimate |
| --- | --- | --- | --- | --- |
| Breast cancer 5 year survival | BETA | 439.69 | 2354.44 | 0.157 |
| Colorectal cancer 5 year survival | BETA | 1457.56 | 1806.35 | 0.447 |

## Osteoarthritis

The parameter distributions for the incidence and hazard ratios for osteoarthritis are reported below.

Table 16: Input parameters for the osteoarthritis risk model (15)

| Parameter Description | Distribution | Parameter 1 | Parameter 2 | Central estimate |
| --- | --- | --- | --- | --- |
| Osteoarthritis incidence | NORMAL | 0.0053 | 0.0000004 | 0.0053 |
| Osteoarthritis RR of diabetes | LOGNORMAL | 0.723 | 0.317 | 2.06 |
| Osteoarthritis RR of BMI | LOGNORMAL | 0.073 | 0.026 | 1.076 |

## Depression

The parameter distributions for the incidence and hazard ratios for depression are reported below.

Table 17: Input parameters for the depression risk model

| Parameter Description | Distribution | Parameter 1 | Parameter 2 | Central estimate | Source |
| --- | --- | --- | --- | --- | --- |
| Odds of depression | BETA | 336 | 8803 | 0.0397 | (16) |
| Odds ratio for diabetes | LOGNORMAL | 0.4187 | 0.1483 | 1.52 | (16) |
| Odds ratio for stroke | LOGNORMAL | 1.8406 | 0.5826 | 6.3 | (17) |

## Mortality

The other cause mortality rates by age were assumed constant in the probabilistic sensitivity analysis (18). The parameter distribution for the hazard ratio for other cause mortality with diabetes is reported below.

Table 18: Input parameters for mortality hazard ratio for diabetes (19)

| Parameter Description | Distribution | Parameter 1 | Parameter 2 | Central estimate |
| --- | --- | --- | --- | --- |
| Mortality hazard ratio for diabetes | LOGNORMAL | 0.588 | 0.186 | 1.80 |

# Utilities

The parameter distributions used to estimate health state utilities in the model are reported below.

Table 19: Utility input parameters

| Parameter Description | Distribution | Parameter 1 | Parameter 2 | Central estimate | Source |
| --- | --- | --- | --- | --- | --- |
| Renal/ulcer baseline utility | NORMAL | 0.689 | 0.014 | 0.689 | (20) |
| Renal dialysis | NORMAL | -0.078 | 0.026 | -0.078 | (20) |
| Foot ulcer | NORMAL | -0.099 | 0.013 | -0.099 | (20) |
| Amputation/heart failure baseline utility | NORMAL | 0.807 | 0.005 | 0.807 | (8) |
| Heart failure | NORMAL | -0.101 | 0.032 | -0.101 | (8) |
| Amputation | NORMAL | -0.172 | 0.045 | -0.172 | (8) |
| Stable angina multiplicative factor decrement | NORMAL | 0.801 | 0.038 | 0.801 | (5) |
| Unstable angina multiplicative factor decrement | NORMAL | 0.77 | 0.038 | 0.77 | (5) |
| MI multiplicative factor decrement | NORMAL | 0.76 | 0.018 | 0.76 | (5) |
| Stroke multiplicative factor decrement | NORMAL | 0.629 | 0.04 | 0.629 | (5) |
| Cancer baseline utility | NORMAL | 0.8 | 0.0026 | 0.8 | (21) |
| Cancer decrement | NORMAL | -0.06 | 0.008 | -0.06 | (21) |
| Osteoarthritis utility | NORMAL | 0.69 | 0.069 | 0.69 | (22) |
| Depression baseline utility | NORMAL | 0.48 | 0.048 | 0.48 | (23) |
| Depression remitters | NORMAL | 0.31 | 0.031 | 0.31 | (23) |
| Depression responders | NORMAL | 0.20 | 0.020 | 0.20 | (23) |
| Depression non-responders | NORMAL | 0.070 | 0.007 | 0.070 | (23) |
| Depression drop-outs | NORMAL | 0.050 | 0.005 | 0.050 | (23) |
| Weight loss utility decrement | NORMAL | -0.0025 | 0.001 | -0.0025 | (24;25) |
| Age utility decrement | NORMAL | -0.004 | 0.0001 | -0.004 | (5) |

# Unit Health Care Costs

| Parameter Description | Distribution | Parameter 1 | Parameter 2 | Central estimate | Source |
| --- | --- | --- | --- | --- | --- |
| Cost of insulin | GAMMA | 3.367 | 408.6 | 1375.72 | (26) |
| Cost of anti-hypertensives | GAMMA | 100 | 1.96 | 195.94 | (27) |
| Cost of GP appointment | GAMMA | 100 | 0.47 | 46.95 | (28) |
| Nurse appointment (Advanced) | GAMMA | 100 | 0.26 | 25.52 | (28) |
| Health care assistant appointment | GAMMA | 100 | 0.03 | 3.40 | (28) |
| Eye screening | GAMMA | 15.3664 | 1.58219 | 24.31 | (29) |
| HbA1c test | GAMMA | 100 | 0.03 | 3.00 | (30) |
| Lipids test | GAMMA | 100 | 0.01 | 1.00 | (30) |
| LfT test | GAMMA | 100 | 0.01 | 1.00 | (30) |
| B12 test | GAMMA | 100 | 0.01 | 1.00 | (30) |
| Urine test | GAMMA | 100 | 0.01 | 1.00 | (30) |
| Nicotine replacement therapy | GAMMA | 100 | 1.03 | 103.00 | (28) |
| HbA1c diagnosis screening | GAMMA | 100 | 0.148147 | 14.81 | (30) |
| Unstable Angina hospital admission | GAMMA | 100 | 12.75591 | 1275.59 | (3) |
| Revascularisation in hospital | GAMMA | 100 | 60.36846 | 6036.85 | (3) |
| MI Hospital admission | GAMMA | 100 | 15.54896 | 1554.90 | (3) |
| First Outpatient appointment | GAMMA | 100 | 1.653571 | 165.36 | (3) |
| Subsequent outpatient appointments | GAMMA | 100 | 1.100574 | 110.06 | (3) |
| Fatal CHD | GAMMA | 100 | 7.125001 | 712.50 | (31) |
| Fatal Stroke | GAMMA | 100 | 44.42562 | 4442.56 | (32) |
| First year stroke cost | GAMMA | 100 | 126.77 | 12,676.60 | (33) |
| Subsequent year stroke cost | GAMMA | 100 | 17.399 | 1739.91 | (33) |
| Transient Ischemic Attack | GAMMA | 100 | 27.266 | 2722.65 | (33) |
| Glytrin Spray | CONSTANT | NA | NA | 12.61 | (3) |
| Isosorbide mononitrate | CONSTANT | NA | NA | 13.54 | (3) |
| Verapamil | CONSTANT | NA | NA | 50.57 | (3) |
| Atenolol | CONSTANT | NA | NA | 36.42 | (3) |
| Aspirin | CONSTANT | NA | NA | 8.01 | (3) |
| Ramipril | CONSTANT | NA | NA | 90.45 | (3) |
| ARB | CONSTANT | NA | NA | 253.28 | (3) |
| Clopidogrel | CONSTANT | NA | NA | 554.41 | (3) |
| Congestive Heart Failure inpatient year 1 | GAMMA | 17.088 | 197.61 | 3376.7 | (34) |
| Congestive Heart Failure non-inpatient year 1 | GAMMA | 50.135 | 20.664 | 1,035.97 | (34) |
| Congestive Heart Failure inpatient subsequent | GAMMA | 23.465 | 66.426 | 1558.71 | (34) |
| Congestive Heart Failure non-inpatient subsequent | GAMMA | 109.8 | 9.377 | 1,029.62 | (34) |
| Blindness inpatient year 1 | GAMMA | 7.98 | 179.63 | 1433.85 | (34) |
| Blindness non-inpatient year 1 | GAMMA | 14.799 | 127.99 | 1894.16 | (34) |
| Blindness inpatient subsequent years | GAMMA | 41.395 | 11.58 | 479.36 | (34) |
| Blindness non-inpatient subsequent years | GAMMA | 79.725 | 9.7955 | 780.94 | (34) |
| Amputation inpatient year 1 | GAMMA | 35.733 | 282.7 | 1896.28 | (34) |
| Amputation non-inpatient year 1 | GAMMA | 16.817 | 169.84 | 2856.05 | (34) |
| Amputation inpatient subsequent years | GAMMA | 23.023 | 82.364 | 1792 | (34) |
| Amputation non-inpatient subsequent years | GAMMA | 57.062 | 29.875 | 1611 | (34) |
| Renal Haemodialysis | GAMMA | 100 | 420.49 | 42049.00 | (35) |
| Renal Automated Peritoneal dialysis | GAMMA | 100 | 272.1714 | 27217.14 | (35) |
| Renal Ambulatory peritoneal dialysis | GAMMA | 100 | 197.4225 | 19742.25 | (35) |
| Renal transplant | GAMMA | 100 | 236.5973 | 23659.73 | (36) |
| Immunosuppressants | GAMMA | 100 | 69.58745 | 6958.75 | (36) |
| Foot ulcer not infected | GAMMA | 100 | 1.677526 | 167.75 | (37) |
| Foot ulcer with cellulitis | GAMMA | 100 | 4.431003 | 443.10 | (37) |
| Foot ulcer with osteomyelitis | GAMMA | 100 | 8.215817 | 821.58 | (37) |
| Breast Cancer | GAMMA | 100 | 138.1811 | 13818.11 | (38) |
| Colorectal cancer Dukes A | GAMMA | 100 | 100.9135 | 10091.35 | (39) |
| Colorectal cancer Dukes B | GAMMA | 100 | 173.1532 | 17315.32 | (39) |
| Colorectal cancer Dukes C | GAMMA | 100 | 265.5026 | 26550.26 | (39) |
| Colorectal cancer Dukes D | GAMMA | 100 | 166.2553 | 16625.53 | (39) |
| Osteoarthritis | GAMMA | 100 | 9.616886 | 961.69 | (40) |
| Depression – Practice nurse surgery | GAMMA | 100 | 0.090154 | 9.02 | (41) |
| Depression – Practice nurse home | GAMMA | 100 | 0.270463 | 27.05 | (41) |
| Depression – Practice nurse telephone | GAMMA | 100 | 0.090154 | 9.02 | (41) |
| Depression – Health visitor | GAMMA | 100 | 0.387834 | 38.78 | (41) |
| Depression – District nurse | GAMMA | 100 | 0.377628 | 37.76 | (41) |
| Depression – Other nurse | GAMMA | 100 | 0.090154 | 9.02 | (41) |
| Depression – HCA phlebotomist | GAMMA | 100 | 0.034021 | 3.40 | (41) |
| Depression – Other primary care | GAMMA | 100 | 0.255154 | 25.52 | (41) |
| Depression – Out of Hours | GAMMA | 100 | 0.268661 | 26.87 | (41) |
| Depression – NHS Direct | GAMMA | 100 | 0.25295 | 25.30 | (41) |
| Depression – Walk-in Centre | GAMMA | 100 | 0.388316 | 38.83 | (41) |
| Depression – Prescribed medicines | GAMMA | 100 | 0.096144 | 9.61 | (41) |
| Depression – Secondary Care | GAMMA | 100 | 0.81 | 81.00 | (41) |

Reference List

(1) Green MA, Li J, Relton C, Strong M, Kearns B, Wu M, et al. Cohort Profile: The Yorkshire Health Study. Int J Epidemiol 2014 Jul 9;dyu121.

(2) Clarke PM, Gray AM, Briggs A, Farmer AJ, Fenn P, Stevens RJ, et al. A model to estimate the lifetime health outcomes of patients with type 2 diabetes: the United Kingdom Prospective Diabetes Study (UKPDS) Outcomes Model (UKPDS no. 68). Diabetologia 2004 Oct;47(10):1747-59.

(3) Ara R, Pandor A, Stevens J, Rees A, Rafia R. Early high-dose lipid-lowering therapy to avoid cardiac events: a systematic review and economic evaluation. Health Technol Assess 2009 Jul;13(34):1-118.

(4) Wald DS, Law M, Morris JK, Bestwick JP, Wald NJ. Combination therapy versus monotherapy in reducing blood pressure: meta-analysis on 11,000 participants from 42 trials. Am J Med 2009 Mar;122(3):290-300.

(5) Ward S, Lloyd JM, Pandor A, Holmes M, Ara R, Ryan A, et al. A systematic review and economic evaluation of statins for the prevention of coronary events. Health Technol Assess 2007 Apr;11(14):1-iv.

(6) Davies MJ, Heller S, Skinner TC, Campbell MJ, Carey ME, Cradock S, et al. Effectiveness of the diabetes education and self management for ongoing and newly diagnosed (DESMOND) programme for people with newly diagnosed type 2 diabetes: cluster randomised controlled trial. BMJ 2008 Mar 1;336(7642):491-5.

(7) Hippisley-Cox J, Coupland C, Vinogradova Y, Robson J, Minhas R, Sheikh A, et al. Predicting cardiovascular risk in England and Wales: prospective derivation and validation of QRISK2. BMJ 2008 Jun 28;336(7659):1475-82.

(8) Hayes AJ, Leal J, Gray AM, Holman RR, Clarke PM. UKPDS outcomes model 2: a new version of a model to simulate lifetime health outcomes of patients with type 2 diabetes mellitus using data from the 30 year United Kingdom Prospective Diabetes Study: UKPDS 82. Diabetologia 2013 Sep;56(9):1925-33.

(9) Khaw KT, Wareham N, Luben R, Bingham S, Oakes S, Welch A, et al. Glycated haemoglobin, diabetes, and mortality in men in Norfolk cohort of european prospective investigation of cancer and nutrition (EPIC-Norfolk). BMJ 2001 Jan 6;322(7277):15-8.

(10) Kannel WB, D'Agostino RB, Silbershatz H, Belanger AJ, Wilson PW, Levy D. Profile for estimating risk of heart failure. Arch Intern Med 1999 Jun 14;159(11):1197-204.

(11) Pischon T, Lahmann PH, Boeing H, Friedenreich C, Norat T, Tjonneland A, et al. Body size and risk of colon and rectal cancer in the European Prospective Investigation Into Cancer and Nutrition (EPIC). J Natl Cancer Inst 2006 Jul 5;98(13):920-31.

(12) Lahmann PH, Hoffmann K, Allen N, van Gils CH, Khaw KT, Tehard B, et al. Body size and breast cancer risk: findings from the European Prospective Investigation into Cancer And Nutrition (EPIC). Int J Cancer 2004 Sep;111(5):762-71.

(13) Renehan AG, Tyson M, Egger M, Heller RF, Zwahlen M. Body-mass index and incidence of cancer: a systematic review and meta-analysis of prospective observational studies. Lancet 2008 Feb 16;371(9612):569-78.

(14) Cancer Survival in England: Patients Diagnosed, 2006–2010 and Followed up to 2011. Office of National Statistics 2012Available from: URL: <http://www.ons.gov.uk/ons/publications/re-reference-tables.html?edition=tcm%3A77-277733>

(15) Schett G, Kleyer A, Perricone C, Sahinbegovic E, Iagnocco A, Zwerina J, et al. Diabetes is an independent predictor for severe osteoarthritis: results from a longitudinal cohort study. Diabetes Care 2013 Feb;36(2):403-9.

(16) Golden SH, Lazo M, Carnethon M, Bertoni AG, Schreiner PJ, Diez Roux AV, et al. Examining a bidirectional association between depressive symptoms and diabetes. JAMA 2008 Jun 18;299(23):2751-9.

(17) Whyte EM, Mulsant BH, Vanderbilt J, Dodge HH, Ganguli M. Depression after stroke: a prospective epidemiological study. J Am Geriatr Soc 2004 May;52(5):774-8.

(18) Mortality Statistics: Deaths registered in England and Wales (Series DR), 2011. Office of National Statistics 2013Available from: URL: <http://www.ons.gov.uk/ons/publications/re-reference-tables.html?edition=tcm%3A77-277727>

(19) Seshasai SR, Kaptoge S, Thompson A, Di AE, Gao P, Sarwar N, et al. Diabetes mellitus, fasting glucose, and risk of cause-specific death. N Engl J Med 2011 Mar 3;364(9):829-41.

(20) Coffey JT, Brandle M, Zhou H, Marriott D, Burke R, Tabaei BP, et al. Valuing health-related quality of life in diabetes. Diabetes Care 2002 Dec;25(12):2238-43.

(21) Yabroff KR, Lawrence WF, Clauser S, Davis WW, Brown ML. Burden of illness in cancer survivors: findings from a population-based national sample. J Natl Cancer Inst 2004 Sep 1;96(17):1322-30.

(22) Black C, Clar C, Henderson R, MacEachern C, McNamee P, Quayyum Z, et al. The clinical effectiveness of glucosamine and chondroitin supplements in slowing or arresting progression of osteoarthritis of the knee: a systematic review and economic evaluation. Health Technol Assess 2009 Nov;13(52):1-148.

(23) Benedict A, Arellano J, De CE, Baird J. Economic evaluation of duloxetine versus serotonin selective reuptake inhibitors and venlafaxine XR in treating major depressive disorder in Scotland. J Affect Disord 2010 Jan;120(1-3):94-104.

(24) Warren E, Brennan A, Akehurst R. Cost-effectiveness of sibutramine in the treatment of obesity. Med Decis Making 2004 Jan;24(1):9-19.

(25) O'Meara S, Riemsma R, Shirran L, Mather L, ter Riet G. A rapid and systematic review of the clinical effectiveness and cost-effectiveness of orlistat in the management of obesity. Health Technol Assess 2001;5(18):1-81.

(26) Poole C, Tetlow T, McEwan P, Holmes P, Currie C. The prescription cost of managing people with type 1 and type 2 diabetes following initiation of treatment with either insulin glargine or insulin determir in routine general practice in the UK: a retrospective database analysis. Current Medical Research and Opinion 2007;23(1):S41-S48.

(27) Blak BT, Mullins CD, Shaya FT, Simoni-Wastila L, Cooke CE, Weir MR. Prescribing trends and drug budget impact of the ARBs in the UK. Value Health 2009 Mar;12(2):302-8.

(28) Curtis L. Unit costs of health and social care. 2014.

(29) Burr JM, Mowatt G, Hernandez R, Siddiqui MA, Cook J, Lourenco T, et al. The clinical effectiveness and cost-effectiveness of screening for open angle glaucoma: a systematic review and economic evaluation. Health Technol Assess 2007 Oct;11(41):iii-x, 1.

(30) NHS reference costs 2013-14. Department of Health 2015Available from: URL: https://[www.gov.uk/government/publications/nhs-reference-costs-2013-to-2014](http://www.gov.uk/government/publications/nhs-reference-costs-2013-to-2014)

(31) Palmer S, Sculpher M, Philips Z, Robinsonm M., Ginnelly L, Bakhai A eal. A cost-effectiveness model comparing alternative management strategies for the use of glycoprotein IIb/IIIa antagonists in non-ST-elevation acute coronary syndrome. Report to the National Institute for Clinical Excellence.; 2008.

(32) Youman P, Wilson K, Harraf F, Kalra L. The economic burden of stroke in the United Kingdom. Pharmacoeconomics 2003;21 Suppl 1:43-50.:43-50.

(33) Luengo-Fernandez R, Gray AM, Rothwell PM. A population-based study of hospital care costs during 5 years after transient ischemic attack and stroke. Stroke 2012 Dec;43(12):3343-51.

(34) Alva M, Gray A, Mihaylova B, Leal J, Holman R. The impact of diabetes-related complications on healthcare costs: new results from the UKPDS (UKPDS 84). Diabetic Medicine 2014;459-66.

(35) Baboolal K, McEwan P, Sondhi S, Spiewanowski P, Wechowski J, Wilson K. The cost of renal dialysis in a UK setting--a multicentre study. Nephrol Dial Transplant 2008 Jun;23(6):1982-9.

(36) Cost-effectiveness of transplantation. NHS Blood and Transplant . 2013.

Ref Type: Online Source

(37) Gordois A, Scuffham P, Shearer A, Oglesby A, Tobian JA. The health care costs of diabetic peripheral neuropathy in the US. Diabetes Care 2003 Jun;26(6):1790-5.

(38) Madan J, Rawdin A, Stevenson M, Tappenden P. A rapid-response economic evaluation of the UK NHS Cancer Reform Strategy breast cancer screening program extension via a plausible bounds approach. Value Health 2010 Mar;13(2):215-21.

(39) Tappenden P, Eggington S, Nixon R, Chilcott J, Sakai H, Karnon J. Colorectal cancer screening options appraisal Report to the English Bowel Cancer Screening Working Group. National Health Service 2004Available from: URL: <http://www.cancerscreening.nhs.uk/bowel/scharr.pdf>

(40) The economic costs of arthritis for the UK economy. Oxford Economics 2014Available from: URL: https://[www.oxfordeconomics.com/publication/open/222531](http://www.oxfordeconomics.com/publication/open/222531)

(41) Chalder M, Wiles NJ, Campbell J, Hollinghurst SP, Searle A, Haase AM, et al. A pragmatic randomised controlled trial to evaluate the cost-effectiveness of a physical activity intervention as a treatment for depression: the treating depression with physical activity (TREAD) trial. Health Technol Assess 2012;16(10):1-iv.
